# Individualized number of induction chemotherapy cycles for locoregionally advanced nasopharyngeal carcinoma patients based on early tumor response

**DOI:** 10.1002/cam4.5256

**Published:** 2022-09-20

**Authors:** Yu‐Ting Jiang, Kai‐Hua Chen, Zhong‐Guo Liang, Jie Yang, Song Qu, Ling Li, Xiao‐Dong Zhu

**Affiliations:** ^1^ Department of Radiation Oncology Guangxi Medical University Cancer Hospital Nanning China; ^2^ Key Laboratory of Early Prevention and Treatment for Regional High‐Incidence‐ Tumor Guangxi Medical University, Ministry of Education Nanning China; ^3^ Department of Oncology Affiliated Wuming Hospital of Guangxi Medical University Nanning China

**Keywords:** cycle number, induction chemotherapy, nasopharyngeal carcinoma, tumor response

## Abstract

**Background:**

The optimal number of cycles of induction chemotherapy (IC) in locoregionally advanced nasopharyngeal carcinoma (LANPC) is unclear. We aimed to combine the tumor response during IC and tumor stage to individualize the number of IC cycles.

**Methods:**

Totally, 498 LANPC patients who received IC plus CCRT between 2014 and 2018 were reviewed. Tumor response during IC was used to stratify patients with different risks. All patients were classified into those who received two cycles of IC and those who were treated with three cycles. Propensity score matching methods were performed to compare the treatment efficiency.

**Results:**

After two cycles of IC, 340/498 (68.3%) cases showed complete tumor response (CR)/partial response (PR) and 158 (31.7%) achieved stable disease (SD)/disease progression (PD). Unfavorable responders (SD/PD) exhibited poor survival outcomes. The three‐cycle IC regimen was correlated with better OS and PFS than the two‐cycle regimen for N2‐3 patients in the CR/PR group. However, the use of different IC cycle strategies achieved similar survival outcomes for SD/PD or N0‐1 patients. The incidences of acute toxicities were higher in the IC = 3 group.

**Conclusions:**

Tumor response during IC could be a powerful predictor of LANPC and could be used to guide the individualized number of IC cycles. A three‐cycle IC regimen seemed to be preferable for N2‐3 patients who received CR/PR during IC. However, an additional cycle of IC could not benefit N0‐1 or SD/PD patients, and the optimal treatment strategies for these patients require further consideration.

## Graphical abstract

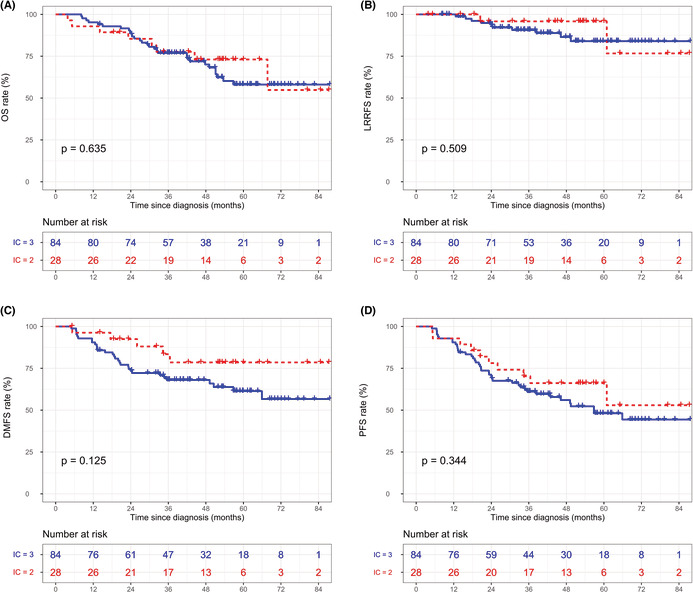

## 1 INTRODUCTION

Nasopharyngeal carcinoma (NPC) is a head and neck malignancy highly prevalent in southeast Asia (especially southern China), eastern Asia, and northern Africa.^1^ As a result of its hidden onset, approximately 70% of the patients are initially defined as locoregionally advanced NPC (LANPC), and concurrent chemoradiotherapy (CCRT) is the main treatment for these patients.^2^, ^3^, ^4^ Despite the application of intensity‐modulated radiotherapy (IMRT) and systemic chemotherapy, management of LANPC remains a challenge, as nearly 30% of patients develop disease progression after treatment.^4^ The results from some well‐designed randomized controlled trials indicated that using induction chemotherapy (IC) before CCRT provided survival benefits for LANPC patients.^5^, ^6^, ^7^, ^8^ Guidelines of the NCCN and CSCO both strongly recommend IC combined with CCRT for LANPC,^9^, ^10^ which has promoted its widespread use in clinical practice.

A majority of clinical trials usually apply three cycles of IC as the primary strategy.^8^, ^11^, ^12^, ^13^ However, it is undetermined whether three cycles produce the greatest survival benefit. Between 4% and 11% of patients did not complete three IC cycles because of treatment‐associated adverse events and cost. Our previous study demonstrated that the two‐cycle IC regimen achieved similar long‐term survival outcomes compared with the three‐cycle regimen and had fewer treatment‐related acute toxicities, which is consistent with the results of several other retrospective studies.^14^, ^15^, ^16^ Although IC is a highly effective treatment strategy for LANPC, there are still some patients with poor sensitivity to chemotherapy, who experience tumor responses of stable disease (SD) or progressive disease (PD). Several previous studies demonstrated that patients who showed a tumor response of SD/PD after IC had a worse survival than patients who had complete response (CR)/partial response (PR).^17^, ^18^, ^19^ Considering the significant difference in survival between patients with good and poor tumor responses, it seems reasonable to provide individualized treatment regimens. In clinical practice, patients are generally reviewed after two cycles of IC to evaluate the treatment efficiency. To the best of our knowledge, no study has explored the relationship between an early tumor response during IC and the optimal number of IC cycles. While more convincing evidence is awaited, it is necessary to optimize patient selection for the number of IC cycles to ensure therapeutic efficacy while avoiding excessive toxicity.

We conducted this study to explore the treatment efficiencies of different IC cycle regimens for LANPC based on the tumor response during IC. Our findings will aid oncologists in predicting the outcome of NPC, and they will guide strategies for selecting the optimal number of IC cycles.

## 2 MATERIALS AND METHODS

### 2.1 Patients

Between January 2014 and June 2018, 498 newly diagnosed NPC patients were reviewed. The entry criteria were as follows: (1) histologically proven NPC with stage III or IVA disease (8th AJCC staging system); (2) in receipt of two or three cycles of IC and CCRT; (3) available magnetic resonance imaging (MRI) information before treatment and after two cycles of IC; (4) complete clinical and follow‐up information; (5) without other malignancy, pregnancy, and lactation; and (6) adequate organ function. The institutional review board of our center approved the current study, and the necessity for informed consent was waived.

### 2.2 Treatment

All patients were treated based on the NPC treatment principles of our institute and the general situation of patients. All IC regimens were given based on platinum anticancer drugs, including TPF, TP, PF, and GP. The concurrent chemotherapy was the cisplatin regime. Radiation was delivered by IMRT. Details of treatment have been described previously and described in the Appendix S2.^20^

### 2.3 Tumor response evaluation

Each patient underwent a nasopharyngeal and neck MRI before and after the two cycles of IC. The tumor response was analyzed by two independent clinicians in a double‐blinded manner according to the Response Evaluation Criteria in Solid Tumors criteria 1.1 (RECIST 1.1),^21^ which divided patients into four grades (CR, PR, SD, and PD) (see Appendix S2). Tumor responses in the current study were segmented into two classes: “favorable responders” (CR/PR) and “unfavorable responders” (SD/PD).

### 2.4 Follow‐up and endpoints

Routine examinations were performed for patients once every 3 months in the first 2 years, once every 6 months for the next 2 years, and once afterward. If the patient did not return to the hospital for review, the patient or their family members will be followed up by telephone. OS served as the main endpoint, which was assigned as the time from the diagnosis to death from any cause. Other endpoints included LRRFS, DMFS, and PFS, which measured the time from diagnosis to first locoregional relapse, distant metastasis, and any failure or death. The grade of acute hematological toxicities during IC was determined based on the Common Terminology Criteria for Adverse Events (version 3.0).

### 2.5 Statistical analysis

Continuous variables were converted into categorical variables and presented using frequencies and percentages. Categorical data in the two groups were compared by the chi‐square test or Fisher's exact test. One‐to‐one and many‐to‐one propensity score matching (PSM) methods were employed to minimize the influence of confounding factors in comparing the treatment efficiency.^22^, ^23^ The survival rates were estimated using the Kaplan‐Meier method with the log‐rank test. Multivariable Cox regression analysis was carried out to select independent prognostic factors from among those with *p* < 0.10 in the univariable analysis. All statistical analyses were conducted using SPSS 22.0 and R 3.6.0. Unless otherwise noted, a two‐sided *p* value <0.05 was regarded as significant.

## 3 RESULTS

### 3.1 Baseline characteristics

In all 498 patients, 340 (68.3%) patients received CR/PR after two cycles of IC, and 158 (31.7%) patients had SD/PD. First, we divided the entire cohort into “CR/PR” and “SD/PD” groups. The baseline characteristics of all patients with different tumor responses stratified by the number of IC cycles are shown in Table S1. In different tumor response groups, the two‐cycle IC strategy was applied less frequently than the three‐cycle strategy (“CR/PR” group: 45, 13.2% vs. 295, 86.8%; “SD/PD” group: 28, 17.7% vs. 130, 82.3%). The correlation analysis indicated that no significant association was observed between the number of IC cycles and tumor response (*p* = 0.292). Compared to patients with early clinical stage (stage III), stage IV patients receiving three cycles of IC accounted for a greater proportion (“CR/PR” group: 192, 65.1% vs. 103, 34.9%; “SD/PD” group: 98, 75.4% vs. 32, 24.6%; *p* = 0.046, 0.064, respectively). In addition, there was a statistically significant difference among the different IC cycle groups in regard to the IC regimen (both *p* < 0.005). After one‐to‐two and one‐to‐three PSMs were conducted, baseline characteristics were balanced among the different IC cycle groups in the two tumor response dataset with all p‐values over 0.05 (Table 1).

**TABLE 1:** Basic characteristics of the patients in “CR/PR” and “SD/PD” groups stratified by IC cycles after PSM.

Characteristic	CR/PR (*n* = 135)			SD/PD (*n* = 112)		
	IC = 2	IC = 3	*p*‐value	IC = 2	IC = 3	*p*‐value
Total	45	90		28	84	
Sex			0.349			0.590
Female	14 (31.1)	36 (40.0)		7 (25.0)	16 (19.0)	
Male	31 (68.9)	54 (60.0)		21 (75.0)	68 (81.0)	
Age (years)			1.000			1.000
≤50	17 (37.8)	33 (36.7)		12 (42.9)	36 (42.9)	
>50	28 (62.2)	57 (63.3)		16 (57.1)	48 (57.1)	
Smoking status			0.676			0.809
No	33 (73.3)	69 (76.7)		20 (71.4)	62 (73.8)	
Yes	12 (26.7)	21 (23.3)		8 (28.6)	22 (26.2)	
Histology			0.625			1.000
WHO II	6 (13.3)	16 (17.8)		3 (10.7)	8 (9.5)	
WHO III	39 (86.7)	74 (82.2)		25 (89.3)	76 (90.5)	
T stage			0.301			0.985
T1	0 (0)	1 (1.1)		1 (3.6)	2 (2.4)	
T2	16 (35.6)	28 (31.1)		6 (21.4)	18 (21.4)	
T3	18 (40.0)	26 (28.9)		9 (32.1)	29 (34.5)	
T4	11 (24.4)	35 (38.9)		12 (42.9)	35 (41.7)	
N stage			0.889			0.670
N0	1 (2.2)	3 (3.3)		0 (0)	2 (2.4)	
N1	12 (26.7)	29 (32.2)		12 (42.9)	32 (38.1)	
N2	20 (44.4)	36 (40.0)		10 (35.7)	25 (29.8)	
N3	12 (26.7)	22 (24.4)		6 (21.4)	25 (29.8)	
Overall stage (8th edition)			0.362			0.508
III	23 (51.1)	38 (42.2)		12 (42.9)	30 (35.7)	
IVA	22 (48.9)	52 (57.8)		16 (57.1)	54 (64.3)	
IC regimen			0.084			0.103
TPF	37 (82.2)	78 (86.7)		19 (69.7)	65 (77.4)	
TP	3 (6.7)	6 (6.7)		4 (14.3)	5 (6.0)	
PF	5 (11.1)	2 (2.2)		5 (17.9)	7 (8.3)	
GP	0 (0)	4 (4.4)		0 (0)	7 (8.3)	
Concurrent chemotherapy cycles			0.435			1.000
2	37 (82.2)	79 (87.8)		18 (64.3)	55 (65.5)	
3	8 (17.8)	11 (12.2)		10 (35.7)	29 (34.5)	

*Note*: Data are shown as number of patients (%).

Abbreviations: CR, complete response; GP, gemcitabine‐cisplatin; IC, induction chemotherapy; PD, disease progression; PF, cisplatin‐5‐fluorouracil; PR, partial response; PSM, propensity score matching; SD, stable disease; TP, docetaxel‐cisplatin; TPF, docetaxel‐cisplatin‐5‐fluorouracil; WHO, World Health Organization.

The median follow‐up was 48.1 months (range, 3.8–93.5 months) in the whole cohort. In total, 34 patients (6.8%) had locoregional relapse, 89 (17.9%) experienced distant metastasis, and 105 (21.1%) died.

### 3.2 Survival outcomes

**FIGURE 1:**
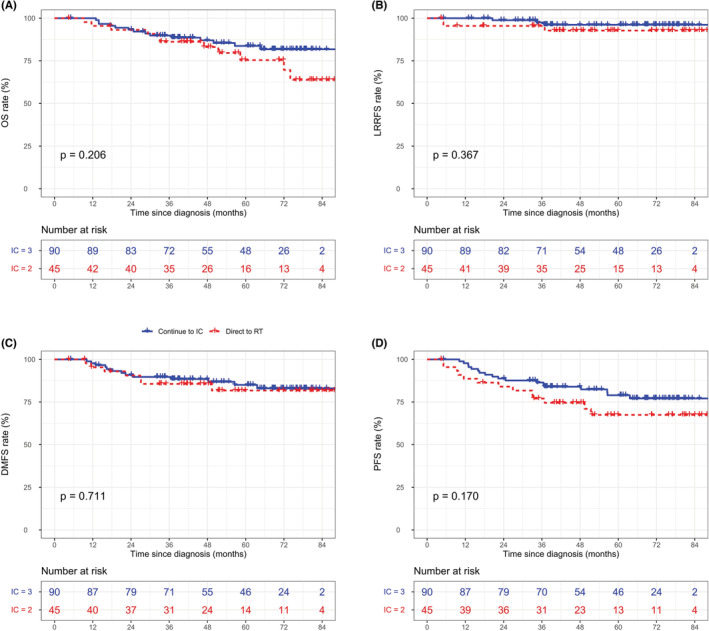
Kaplan‐Meier curves for OS (A), LRRFS (B), DMFS (C), and PFS (D) stratified by IC cycles in the matched “CR/PR” group.

**FIGURE 2:**
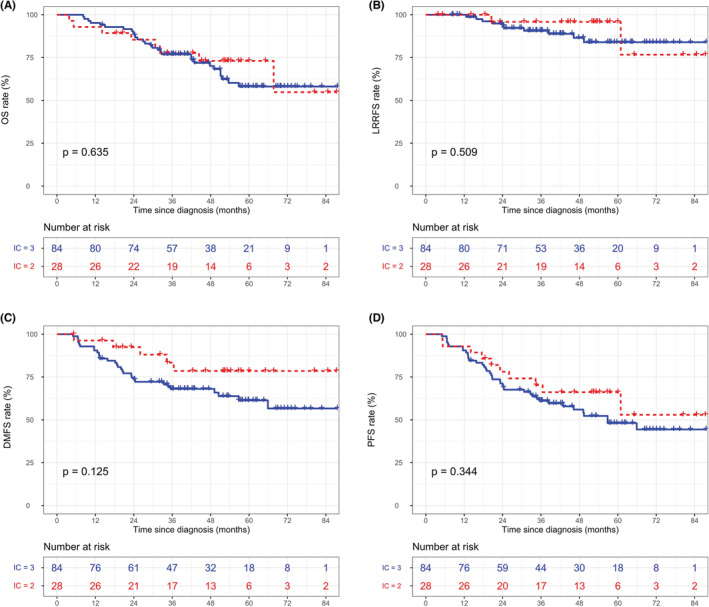
Kaplan‐Meier curves for OS (A), LRRFS (B), DMFS (C), and PFS (D) stratified by IC cycles in the matched “SD/PD” group.

In the whole cohort, the three‐cycle IC regimen was not associated with superior survival outcomes compared with the two‐cycle regimen before (Figure S1) and after (Figure S2) matching. In terms of tumor response during IC, unfavorable responders (SD/PD) showed worse survival rates than favorable responders (CR/PR). The specific data are as follows: (5‐year OS: 60.6% vs. 84.0%, *p* < 0.001, Figure S3A; 5‐year LRRFS: 94.3% vs. 85.1%, *p* < 0.001, Figure S3B; 5‐year DMFS: 85.0% vs. 67.7%, *p* < 0.001, Figure S3C; 5‐year PFS: 76.4% vs. 49.7%, *p* < 0.001, Figure S3D). Thus, after matching with some potential prognostic factors, we further explored the efficiency differences between the two‐ and three‐cycle IC strategies in the different tumor response groups. Detailed survival curves of the matched groups with different tumor responses are depicted in Figure 1 and Figure 2. Briefly, after matching, none of the endpoints reached statistical significance among the different IC cycle groups (all *p* > 0.05). However, it is worth mentioning that for “CR/PR” patients, the 5‐year PFS rate showed a trend to be significantly worse in the IC = 2 group than in the IC = 3 group (67.4% vs. 77.1%, *p* = 0.170, Figure 1D), which was in contrast to the trend of patients in the “SD/PD” group for the 5‐year DMFS rate (78.5% vs. 61.4%, *p* = 0.125, Figure 2C). Table S2 lists the survival rates by IC cycles in the different cohorts.

We included some clinicopathologic variables in the univariable analysis (Table S3). From the results of the univariable analysis in the whole cohort, the following variables were involved in the multivariable analysis of the CR/PR and SD/PD cohorts: age, T stage, N stage, and cycles of IC. Although the IC regimen and concurrent chemotherapy cycles had nonsignificant p‐values for survival outcomes, we still regarded them as potential prognostic factors of survival in this study and included them in the multivariate Cox regression analysis. The results of the Cox regression analyses were similar to those of the log‐rank method, which indicated that the cycles of IC did not be identified as an independent predictor in different tumor response groups in both matched (Table 2 **)** and unmatched cohorts (Table S4). However, in the “CR/PR” group, three cycles of IC showed a tendency to decrease the risk of disease progression (matched cohort: HR: 0.600, 95% CI: 0.293–1.229, *p* = 0.163; unmatched cohort: HR: 0.665, 95% CI: 0.361–1.225, *p* = 0.191).

**TABLE 2:** Multivariable analysis of prognostic factors in matched “CR/PR” and “SD/PD” cohorts.

Characteristic	CR/PR (*n* = 135)		SD/PD (*n* = 122)	
	HR (95% CI)	*p*‐value	HR (95% CI)	*p*‐value
OS				
Age (>50 vs. ≤50)	0.978 (0.423–2.263)	0.959	2.819 (1.273–6.244)	0.011
T stage (T3/4 vs. T1/2)	1.210 (0.481–3.043)	0.686	0.841 (0.386–1.831)	0.663
N stage (N2/3 vs. N0/1)	1.998 (0.717–5.566)	0.185	1.754 (0.763–4.033)	0.186
IC cycles (3 vs. 2)	0.616 (0.279–1.360)	0.231	1.083 (0.489–2.401)	0.844
IC regimen (TPF vs. others)	0.665 (0.220–2.005)	0.469	1.289 (0.582–2.852)	0.531
Concurrent chemotherapy cycles (3 vs. 2)	0.928 (0.268–3.211)	0.906	1.683 (0.858–3.304)	0.130
LRRFS				
Age (>50 vs. ≤50)	0.674 (0.133–3.696)	0.674	1.263 (0.350–4.556)	0.722
T stage (T3/4 vs. T1/2)	0.380 (0.038–3.797)	0.410	0.477 (0.101–2.262)	0.351
N stage (N2/3 vs. N0/1)	0.598 (0.053–6.801)	0.678	0.758 (0.177–3.238)	0.709
IC cycles (3 vs. 2)	0.524 (0.103–2.675)	0.437	1.707 (0.371–7.856)	0.492
IC regimen (TPF vs. others)	0.690 (0.074–6.408)	0.744	1.738 (0.373–8.107)	0.482
Concurrent chemotherapy cycles (3 vs. 2)	1.127 (0.118–10.794)	0.918	1.538 (0.473–5.003)	0.474
DMFS				
Age (>50 vs. ≤50)	0.588 (0.236–1.464)	0.254	1.306 (0.618–2.761)	0.485
T stage (T3/4 vs. T1/2)	2.420 (0.846–6.919)	0.099	0.544 (0.240–1.235)	0.146
N stage (N2/3 vs. N0/1)	3.729 (1.131–12.292)	0.031	1.683 (0.704–4.023)	0.242
IC cycles (3 vs. 2)	0.855 (0.340–2.153)	0.740	1.972 (0.760–5.115)	0.163
IC regimen (TPF vs. others)	3.033 (0.397–23.153)	0.285	1.094 (0.468–2.560)	0.835
Concurrent chemotherapy cycles (3 vs. 2)	0.970 (0.280–3.353)	0.961	1.941 (0.978–3.853)	0.058
PFS				
Age (>50 vs. ≤50)	0.609 (0. 294–1.260)	0.181	1.844 (0.974–3.489)	0.060
T stage (T3/4 vs. T1/2)	1.458 (0.634–3.356)	0.375	0.745 (0.364–1.523)	0.419
N stage (N2/3 vs. N0/1)	2.545 (0.997–6.497)	0.051	1.302 (0.657–2.580)	0.450
IC cycles (3 vs. 2)	0.600 (0.293–1.229)	0.163	1.355 (0.672–2.732)	0.396
IC regimen (TPF vs. others)	0.785 (0.294–2.093)	0.629	0.988 (0.506–1.929)	0.972
Concurrent chemotherapy cycles (3 vs. 2)	0.673 (0.200–2.268)	0.523	1.450 (0.807–2.603)	0.214

Abbreviations: CI, confidence interval; CR, complete response; DMFS, distant metastasis‐free survival; HR, hazard ratio; IC, induction chemotherapy; LRRFS, locoregional relapse‐free survival; OS, overall survival; PD, disease progression; PFS, progression‐free survival; PR, partial response; SD, stable disease.

### 3.3 Subgroup analysis

Stratified analyses were performed to further investigate the treatment effect of the number of IC cycle based on the N category in different tumor response groups, as N2‐3 stage patients generally showed a higher risk of treatment failure than N0‐1 stage patients. The baseline characteristics were comparable between different IC cycle groups in N2‐3 (Table S5) and N0‐1 patients (Table S6).

**FIGURE 3:**
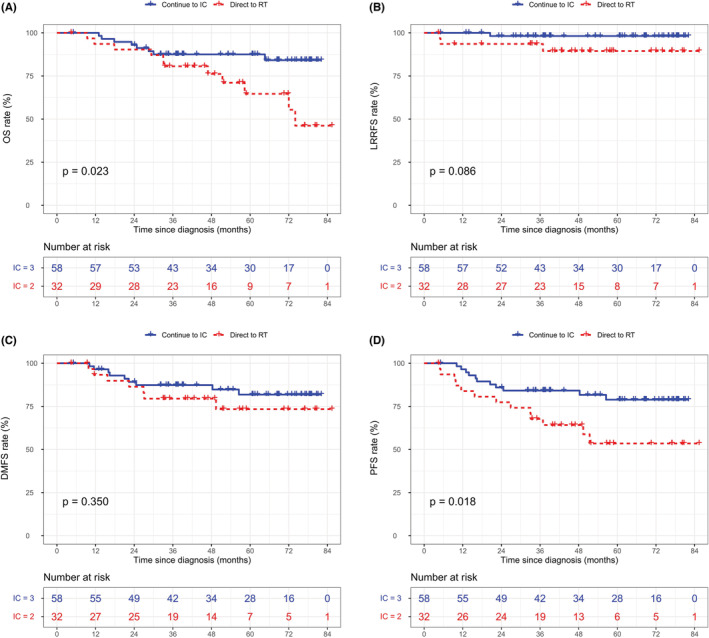
Kaplan‐Meier curves for OS (A), LRRFS (B), DMFS (C), and PFS (D) stratified by IC cycles for N2‐3 patients in the matched “CR/PR” group.

For N2‐3 patients in the “CR/PR” group, those who had a third IC cycle achieved significantly better 5‐year OS and PFS than patients who went directly to radiotherapy (OS: 87.5% vs. 64.6%, *p* = 0.023, Figure 3A; PFS: 78.9% vs. 53.5%, *p* = 0.018, Figure 3D), but not LRRFS (98.5% vs. 89.5%, *p* = 0.086, Figure 3B) and DMFS (81.9% vs. 73.4%, *p* = 0.350, Figure 3C). With regard to N2‐3 patients in the “SD/PD” group, no significant difference in endpoints was observed between the two IC cycle regimen groups (Figure S4). In the multivariable analysis of N2‐3 patients in the “CR/PR” group (Table 3), three‐cycle IC regimen was an independent favorable factor for OS (HR: 0.349, 95% CI: 0.139–0.872, *p* = 0.024) and PFS (HR: 0.370, 95% CI: 0.165–0.833, *p* = 0.016), but not for LRRFS (HR: 0.195, 95% CI: 0.020–1.907, *p* = 0.160) and DMFS (HR: 0.609, 95% CI: 0.224–1.650, *p* = 0.329).

**TABLE 3:** Multivariable analysis of prognostic factors for N2‐3 patients in matched “CR/PR” and “SD/PD” cohorts.

	CR/PR (*n* = 90)		SD/PD (*n* = 67)	
Characteristic	HR(95%CI)	*p*‐value	HR(95%CI)	*p*‐value
OS				
Age (>50 vs. ≤50)	0.710 (0.264–1.909)	0.498	2.841 (0.969–8.327)	0.057
T stage (T3/4 vs. T1/2)	1.392 (0.520–3.726)	0.511	0.994 (0.439–2.253)	0.989
N stage (N3 vs. N2)	1.684 (0.663–4.275)	0.273	1.873 (0.811–4.326)	0.142
IC cycles (3 vs. 2)	0.349 (0.139–0.872)	0.024	0.919 (0.336–2.509)	0.869
IC regimen (TPF vs. others)	0.425 (0.131–1.373)	0.153	1.081 (0.424–2.753)	0.871
Concurrent chemotherapy cycles (3 vs. 2)	1.270 (0.335–4.806)	0.725	1.609 (0.702–3.689)	0.261
LRRFS				
Age (>50 vs. ≤50)	0.649 (0.071–5.922)	0.701	1.164 (0.184–7.366)	0.872
T stage (T3/4 vs. T1/2)	0.375 (0.035–3.993)	0.417	0.616 (0.124–3.065)	0.554
N stage (N3 vs. N2)	0.439 (0.039–4.920)	0.504	1.856 (0.350–9.856)	0.468
IC cycles (3 vs. 2)	0.195 (0.020–1.907)	0.160	1.775 (0.203–15.539)	0.604
IC regimen (TPF vs. others)	0.340 (0.029–3.966)	0.389	NA	0.973
Concurrent chemotherapy cycles (3 vs. 2)	2.482 (0.198–31.090)	0.481	1.492 (0.291–7.645)	0.631
DMFS				
Age (>50 vs. ≤50)	0.390 (0.138–1.104)	0.076	1.236 (0.464–3.294)	0.671
T stage (T3/4 vs. T1/2)	2.909 (0.935–9.049)	0.065	0.671 (0.285–1.580)	0.361
N stage (N3 vs. N2)	1.214 (0.425–3.471)	0.717	2.626 (1.081–6.380)	0.033
IC cycles (3 vs. 2)	0.609 (0.224–1.650)	0.329	1.907 (0.550–6.607)	0.309
IC regimen (TPF vs. others)	2.410 (0.307–18.946)	0.403	0.799 (0.303–2.107)	0.651
Concurrent chemotherapy cycles (3 vs. 2)	1.320 (0.360–4.838)	0.675	1.467 (0.630–3.413)	0.374
PFS				
Age (>50 vs. ≤50)	0.446 (0.191–1.045)	0.063	1.757 (0.719–4.288)	0.216
T stage (T3/4 vs. T1/2)	1.648 (0.675–4.022)	0.273	0.897 (0.424–1.898)	0.777
N stage (N3 vs. N2)	1.235 (0.532–2.864)	0.623	2.270 (1.057–4.877)	0.036
IC cycles (3 vs. 2)	0.370 (0.165–0.833)	0.016	1.015 (0.400–2.578)	0.974
IC regimen (TPF vs. others)	0.561 (0.200–1.572)	0.271	0.714 (0.309–1.649)	0.431
Concurrent chemotherapy cycles (3 vs. 2)	1.027 (0.292–3.613)	0.967	1.556 (0.736–3.288)	0.247

Abbreviations: CI, confidence interval; CR, complete response; DMFS, distant metastasis‐free survival; HR, hazard ratio; IC, induction chemotherapy; LRRFS, locoregional relapse‐free survival; OS, overall survival; PD, disease progression; PFS, progression‐free survival; PR, partial response; SD, stable disease.

For N0‐1 patients, different IC cycle regimens did not exhibit significantly different clinical outcomes among the “CR/PR” (Figure S5) and “SD/PD” groups (Figure S6).

### 3.4 Toxicities

The blood test results during the IC period were collected for patients in the matched “CR/PR” and “SD/PD” groups. Acute toxicities, when most obvious, were comparably evaluated between patients with different numbers of IC cycles (Table 4). Leucopenia was the most common adverse event noted on patients' routine blood tests. The results also showed that the three‐cycle strategy significantly increased the incidence of grade 1–4 anemia (“CR/PR” group: 32.2% vs. 17.8%, *p* = 0.041; “SD/PD” group: 39.3% vs. 10.7%, *p* = 0.005) compared with the two‐cycle strategy in the two tumor response groups and the incidence of grade 1–4 leucopenia (66.7% vs. 39.3%, *p* = 0.014), neutropenia (61.9% vs. 28.6%, *p* = 0.004), and vomiting (70.2% vs. 28.6%, *p* < 0.001) in the “SD/PD” group. No significant differences in other grade 1–4 adverse events such as thrombocytopenia and hepatotoxicity were observed among the different groups. In addition, the intergroup differences in grade 3–4 side effects were not significant.

**TABLE 4:** Treatment‐related adverse events of the patients in matched “CR/PR” and “SD/PD” groups stratified by IC cycles.

Adverse event	CR/PR (*n* = 135)			SD/PD (*n* = 112)		
	IC = 2	IC = 3	*p*‐value	IC = 2	IC = 3	*p*‐value
Total	45	90		28	84	
Leucopenia						
All	30 (66.7)	57 (63.3)	0.848	11 (39.3)	56 (66.7)	0.014
Grade 3–4	5 (11.1)	10 (11.1)	1.000	1 (3.6)	8 (9.5)	0.446
Neutropenia						
All	28 (62.2)	50 (55.6)	0.580	8 (28.6)	52 (61.9)	0.004
Grade 3–4	10 (22.2)	17 (18.9)	0.654	2 (7.1)	13 (10.5)	0.349
Anemia						
All	8 (17.8)	29 (32.2)	0.041	3 (10.7)	33 (39.3)	0.005
Grade 3–4	0 (0)	0 (0)	1.000	0 (0)	0 (0)	1.000
Thrombocytopenia						
All	4 (8.9)	6 (6.7)	0.731	0 (0)	10 (11.9)	0.064
Grade 3–4	1 (2.2)	1 (1.1)	1.000	0 (0)	3 (3.6)	0.572
ALT increase						
All	15 (33.3)	38 (42.2)	0.354	11 (39.3)	30 (35.7)	0.822
Grade 3–4	2 (4.4)	1 (1.1)	0.258	2 (7.1)	2 (3.7)	0.260
AST increase						
All	15 (33.3)	29 (32.2)	1.000	7 (25.0)	25 (29.8)	0.810
Grade 3–4	1 (2.2)	0 (0)	0.333	2 (7.1)	0 (0)	0.057
Bilirubin increase						
All	7 (15.6)	13 (14.4)	1.000	4 (14.3)	19 (22.6)	0.427
Grade 3–4	0 (0)	0 (0)	1.000	0 (0)	0 (0)	1.000
Vomiting						
All	22 (48.9)	52 (57.8)	0.362	8 (28.6)	59 (70.2)	<0.001
Grade 3–4	1 (2.2)	0 (0)	0.333	0 (0)	4 (4.8)	0.570

*Note*: All data are presented as number of patients (%).

Abbreviations: ALT, alanine aminotransferase; AST, aspartate transaminase; CR, complete response; IC, induction chemotherapy; PD, disease progression; PR, partial response; SD, stable disease.

## 4 DISCUSSION

To the best of our knowledge, the present work is the first attempt to individualize the number of IC cycles based on the tumor response during IC in LANPC. Our results revealed that for N2‐3 patients who achieved a tumor response of CR/PR after two cycles of IC, the three‐cycle IC strategy significantly improved survival compared with the two‐cycle strategy, while no significantly different survival outcomes were observed in the subgroups of SD/PD or N0‐1 patients.

The use of IC before radiotherapy confers a survival benefit by eradicating subclinical metastasis. It can also protect adjacent vital organs by shrinking the tumor to reduce the radiation dose to the surrounding tissues.^9^ Although IC plus CCRT is the most recommended regimen according to the latest guidelines for LANPC,^10^ there is still no consensus with respect to the optimal number of cycles of IC. Several previous studies have investigated the prognostic effect of the number of IC cycles for NPC patients and achieved a similar result: more than 2 cycles of IC did not produce a significant survival benefit compared to 2 cycles in the whole cohort.^14^, ^15^, ^16^ One of these studies^16^ reported that in the subgroup of the N2‐3 category, patients who were treated with a two‐cycle IC regimen showed better OS than patients who received 3 or 4 cycles (92.4% vs. 80.8%, *p* = 0.029), which indicated that tumor stage may influence the optimal number of IC cycles in NPC. In contrast, another study suggested that the 4‐cycle IC regimen could improve survival compared with the 2‐cycle regimen in N2‐3 NPC patients.^24^ Our previous study showed similar clinical outcomes between 2‐ and 3‐cycle regimens in subgroup analyses of different tumor stages.^25^ These inconsistent results suggest that the tumor stage alone may not be accurate enough to guide the number of IC cycles. With increasing evidence recommending the addition of IC to CCRT for LANPC patients, the tumor response to IC is attracting increasing attention from oncologists. The use of IC provides an appropriate occasion to assess treatment chemosensitivity that may guide subsequent therapy. Previous studies have demonstrated that the tumor response to IC not only has prognostic effects but also has the potential to guide risk‐adapted chemotherapy before radical radiotherapy.^17^, ^18^, ^26^ The present study also proved a tumor response during IC of “CR/PR” was a favorable predictor for all clinical outcomes. However, it is notable that the impact of the IC cycles was not explored by the subgroup based on the early tumor response during IC. Therefore, we classified patients into two risk groups based on their tumor response during IC and further investigated the individualized number of cycles of IC.

Patients who experienced CR/PR during IC appear to be more sensitive to chemotherapy than those who achieved SD/PD. Although no significant differences were observed, we found that favorable responders in the high cycle group had a trend to exhibit better PFS than patients in the low cycle group. Further subgroup analyses according to the N category showed that the three‐cycle IC strategy significantly improved OS and PFS for CR/PR patients with N2‐3 disease, but not for SD/PD or N0‐1 patients. These results suggest that it is reasonable to combine the tumor response during IC with the N stage to identify candidates suitable for the three‐cycle IC strategy. The N classification is one of the most important variables affecting the selection of chemotherapy. Patients with large and/or extensive lymph node disease (N2‐3) are at high risk. If N2‐3 patients are highly sensitive to chemotherapy, it was hypothesized that increased exposure to effective IC before radiotherapy would reduce the risk of disease progression and lead to survival benefits for these patients. For N0‐1 patients in the CR/PR group, two cycles of IC may be sufficient. Actually, clinical treatment decisions should not only be based on a general assessment of therapeutic efficiency but also take into account the patient's physical condition, economic status, quality of life, and so on. In the current study, we reveal that the three‐cycle IC strategy showed higher incidences of grade 1–4 acute adverse effects, such as gastrointestinal reactions and hematologic toxicities. Although these toxic reactions are uncomplicated and manageable, they may affect subsequent treatment. Regardless, our findings serve as a useful decision aid to guide the application of the third cycle of IC.

For unfavorable responders during IC, these patients were not sensitive to chemotherapy and were generally more likely to experience treatment failures. For this high‐risk subgroup, the additionally ineffective IC would not only fail to confer a survival benefit but would also result in unnecessary side effects and lengthen the waiting time for subsequent radiotherapy, which has been proven to be an adverse predictor.^27^, ^28^, ^29^ The best way to address the above concerns is to identify as precisely as possible those who are most likely to benefit from additional cycles of IC, which will be of great value in the individualized management of NPC patients. Clinicians usually choose a three‐cycle IC regimen as the first option, especially for patients with advanced tumor stage. Nevertheless, our results indicated that different IC cycle strategies achieved similar survival outcomes for SD/PD patients with both early and advanced N stage. Therefore, a three‐cycle regimen is not suitable for unfavorable responders during IC, and applying other more appropriate treatments can be considered for these patients. Targeted therapy and immunotherapy during radiotherapy may be effective treatment choices.^30^, ^31^ An appropriately increasing dose of radiotherapy^7^ or radiotherapy combined with the antitumor drug paclitaxel could be strategies to increase radiation sensitization and are directions for future clinical research. Moreover, in recent years, many molecular mechanisms have been found to be associated with chemotherapy resistance, and some targeted therapies applied to these molecular pathways may provide chemotherapy‐resistant patients with survival benefits.^32^, ^33^, ^34^, ^35^ Although medical centers sometimes apply a reinduction strategy (continuing several cycles of IC with an alternative regimen) to improve the tumor response for patients who initially achieved “SD/PD” to IC, the efficacy of this method is still unclear. A previous study^26^ compared the efficiencies of reinduction therapy and direct radiotherapy on the prognosis of IC‐resistant NPC patients and reported that additional cycles of alternative IC exhibited significantly worse LRRFS and PFS. These results were similar to those of our study, which indicated that continuing IC is not appropriate for NPC patients who show “SD/PD” in response to IC. Since reinduction therapy is not a routine treatment option at our center, further studies in this area have not been explored. Based on the results of this study, direct radiotherapy seemed to be the optimal strategy for LANPC patients who show SD/PD during IC to reduce the waiting time, toxicities, and economic burden.

Currently, there are few high‐quality data directly comparing the efficacy of different IC regimens for LANPC.^36^ TPF, TP, and PF schemes have been applied in our hospital for a long time, and their efficacy in LANPC has been confirmed by some randomized controlled trials.^6^, ^8^, ^11^ GP is a newer regimen of IC showing significant improvements in survival according to large‐scale clinical trials.^13^ Generally, TPF and GP regimens have been considered to be more intensive regimens than other regimens such as PF and TP. Previous scholars have indicated that TPF regimen is superior to PF and TP regimens, whereas is similar to GP regimen in treatment efficiency for LANPC patients.^37^, ^38^, ^39^ However, there was no large‐scale clinical trial with definite results to prove these findings up to now. For LANPC patients treated with CCRT alone, a cumulative dose of cisplatin of 200 mg/m^2^ during radiotherapy is considered to be the optimal dosage.^40^ However, the optimal number of concurrent chemotherapy cycles remains clear for LANPC patients who used IC before CCRT. A retrospective study indicated that two cycles of concurrent chemotherapy achieve an equivalent effect to three cycles for LANPC patients treated with IC and CCRT.^17^ In our study, only 26.7% of patients conducted three cycles of concurrent cisplatin during CCRT. Patient rejection and treatment‐related adverse effects were the most common reasons for failure to continue using the concurrent cisplatin. Many patients have poor physiological status in the late stage of CCRT, and the acute toxicities may reduce patient compliance to the third cycle of concurrent chemotherapy. The current study performed the PSM and multivariable analyses to minimize the influence of different chemotherapy regimens on the study results. More convicting data are expected to determine the optimal IC regimen and concurrent chemotherapy cycles.

The plasma Epstein‐Barr virus (EBV) DNA levels before and during treatment were previously proven to be associated with clinical outcomes.^41^, ^42^, ^43^, ^44^ However, we did not include EBV DNA data in this study. One of the reasons is that in most centers, regular monitoring of EBV DNA is not routine during the treatment of NPC patients. Another consideration is that EBV DNA testing techniques vary widely between different laboratories,^45^ with sensitivity ranging from 53% to 96%,^46^ which greatly affects its clinical application. Therefore, we used the widely accepted RECIST criteria and AJCC TNM staging for IC response and tumor burden evaluation to individualize the cycle number of IC. Further study is necessary to explore the potential of using EBV DNA to guide the decision‐making of the number of IC cycles in NPC.

This study has its limitations. First, inherent biases were unavoidable due to the nature of a retrospective study. Second, the data for analyses were obtained from only one institution with relatively small sample size; therefore, other datasets are needed to validate the results. Third, the clinician's assessment of the patient's disease status and willingness to treat strongly influences the selection of the number of IC cycles, so the potential for selection bias cannot be ignored. In addition, the IC regimens and cycles of concurrent chemotherapy in the current study were not uniform. However, all regimens were recommended by the guidelines and routinely used in our center. Cox regression analysis and the PSM method were used to minimize the influence of biases.

In conclusion, this study demonstrated that tumor response during IC was an independent prognostic factor for LANPC patients. For N2‐3 patients who had CR/PR during IC, the three‐cycle IC regimen significantly improve OS and PFS compared with the two‐cycle regimen. Application of the third IC cycle seemed to be the optimal choice for these patients. However, for N0‐1 or SD/PD patients, three cycles of IC exhibited similar efficacy as two cycles but significantly increased the incidences of acute toxicities. Treatment strategies for these patients deserve further exploration. These findings will aid clinicians in making treatment decisions and provide theoretical a basis for future clinical trials.

## AUTHOR CONTRIBUTIONS

**Yuting Jiang:** Conceptualization (equal); data curation (equal); formal analysis (equal); funding acquisition (equal); investigation (equal); methodology (equal); project administration (equal); resources (equal); software (equal); supervision (equal); validation (equal); visualization (equal); writing – original draft (equal). **Kaihua Chen:** Data curation (equal); formal analysis (equal); funding acquisition (equal); investigation (equal); methodology (equal); project administration (equal); resources (equal); software (equal); supervision (equal); validation (equal); visualization (equal); writing – original draft (equal). **Zhongguo Liang:** Data curation (equal); formal analysis (equal); funding acquisition (equal); investigation (equal); methodology (equal); project administration (equal). **Jie Yang:** Data curation (equal); formal analysis (equal); funding acquisition (equal); investigation (equal); methodology (equal); project administration (equal). **Song Qu:** Resources (equal); software (equal); supervision (equal); validation (equal); visualization (equal); writing – review and editing (equal). **ling Li:** Resources (equal); software (equal); supervision (equal); validation (equal); visualization (equal); writing – review and editing (equal). **Xiaodong Zhu:** Conceptualization (equal); data curation (equal); formal analysis (equal); funding acquisition (equal); investigation (equal); methodology (equal); project administration (equal); resources (equal); software (equal); supervision (equal); validation (equal); visualization (equal); writing – review and editing (equal).

## ETHICS STATEMENT

The present study was approved by the Clinical Research Ethics Committee of Guangxi Medical University Cancer Hospital.

## Supporting information


Appendix S1

Appendix S2

## Data Availability

The data that support the findings of this study are available upon reasonable request.
