# Supplementary figures and images for: Individualized number of induction chemotherapy cycles for locoregionally advanced nasopharyngeal carcinoma patients based on early tumor response
Source: Cancer Med. 2022 Sep 20;12(4):4010–22. doi: 10.1002/cam4.5256 (PMC9972137; doi:10.1002/cam4.5256)

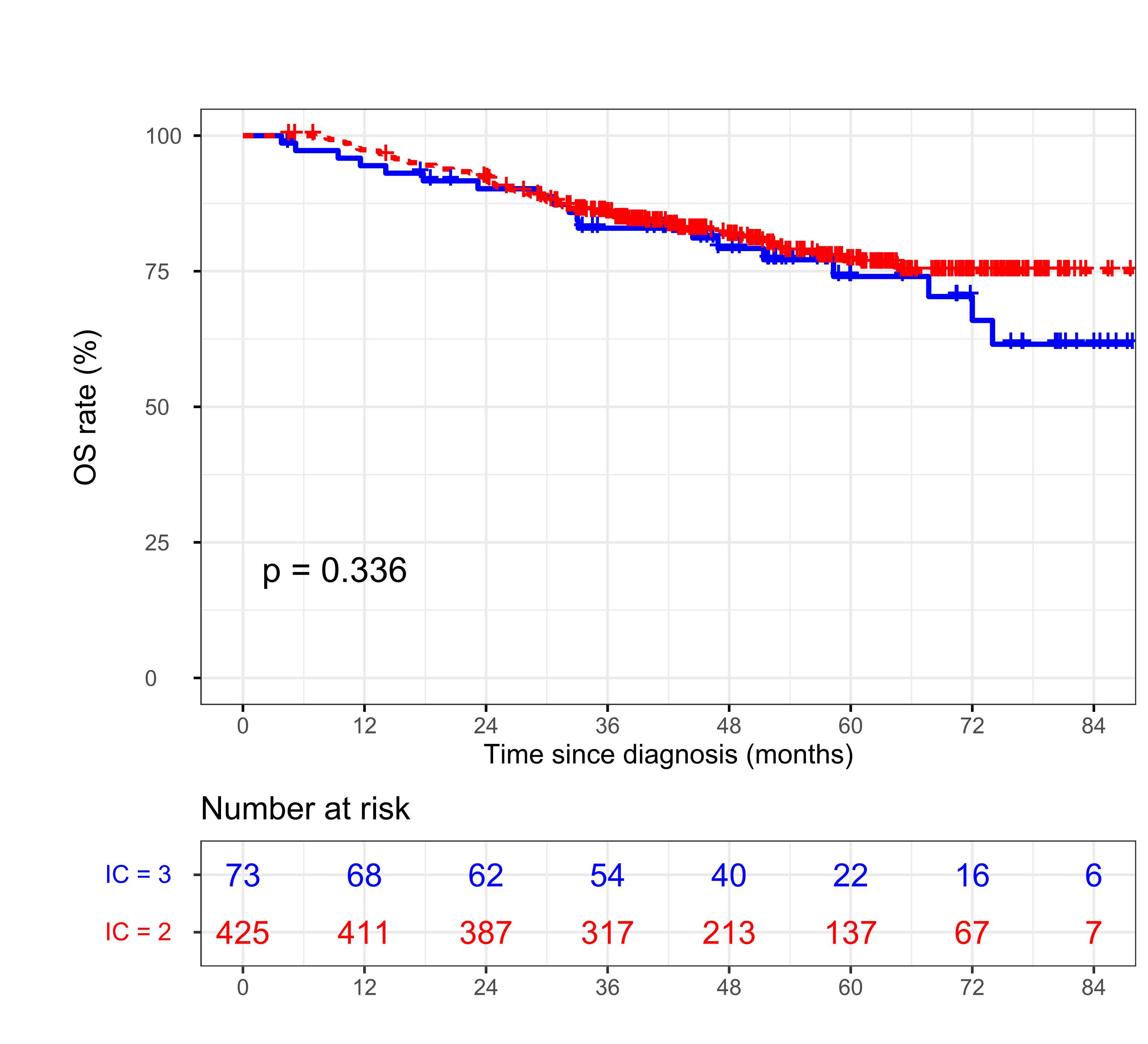

Supplement: Supplementary file 1 — Appendix S1 [file CAM4-12-4010-s001.zip › cam45256-sup-0001-AppendixS1/CAM4_5256_Figure S1A.Tiff]

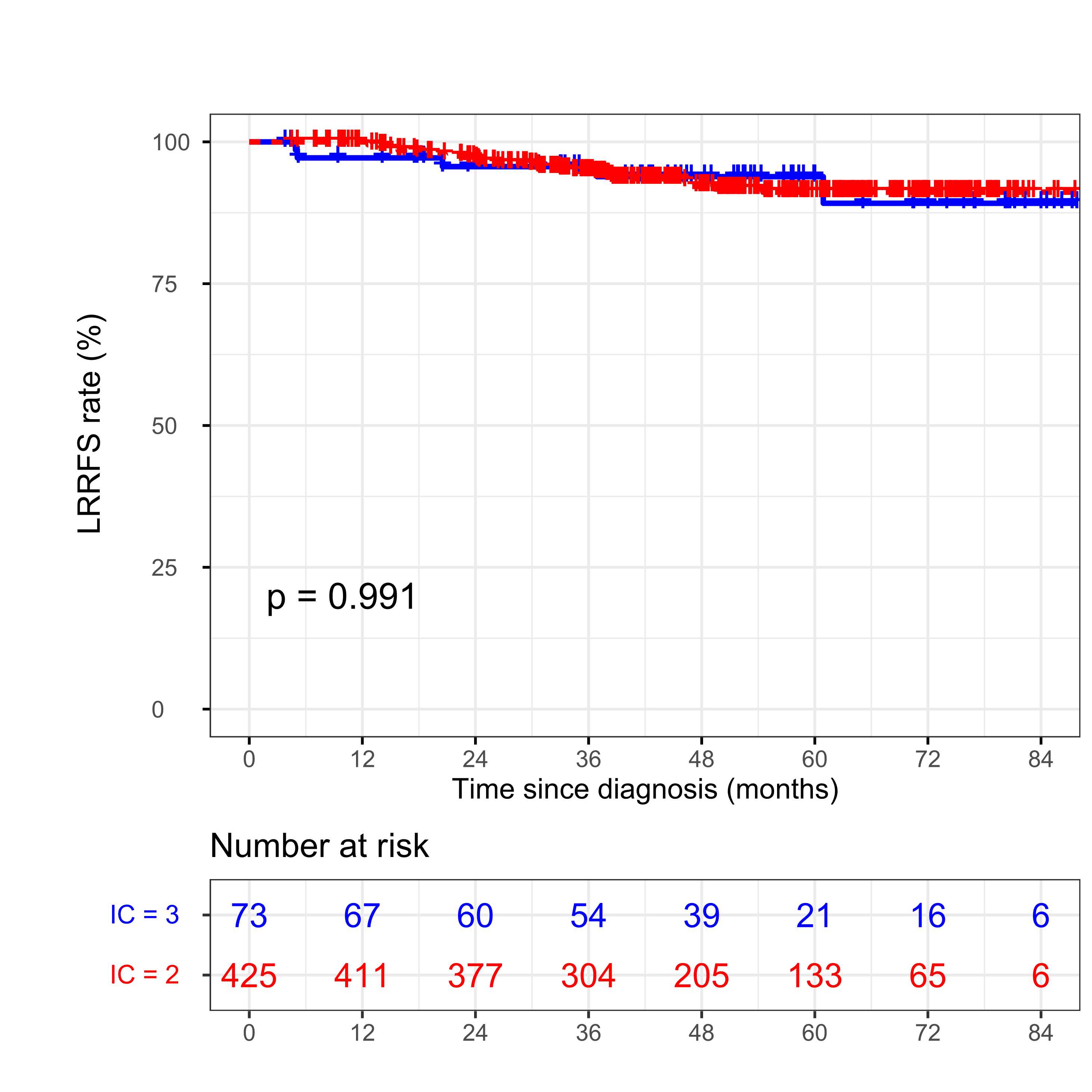

Supplement: Supplementary file 1 — Appendix S1 [file CAM4-12-4010-s001.zip › cam45256-sup-0001-AppendixS1/CAM4_5256_Figure S1B.Tiff]

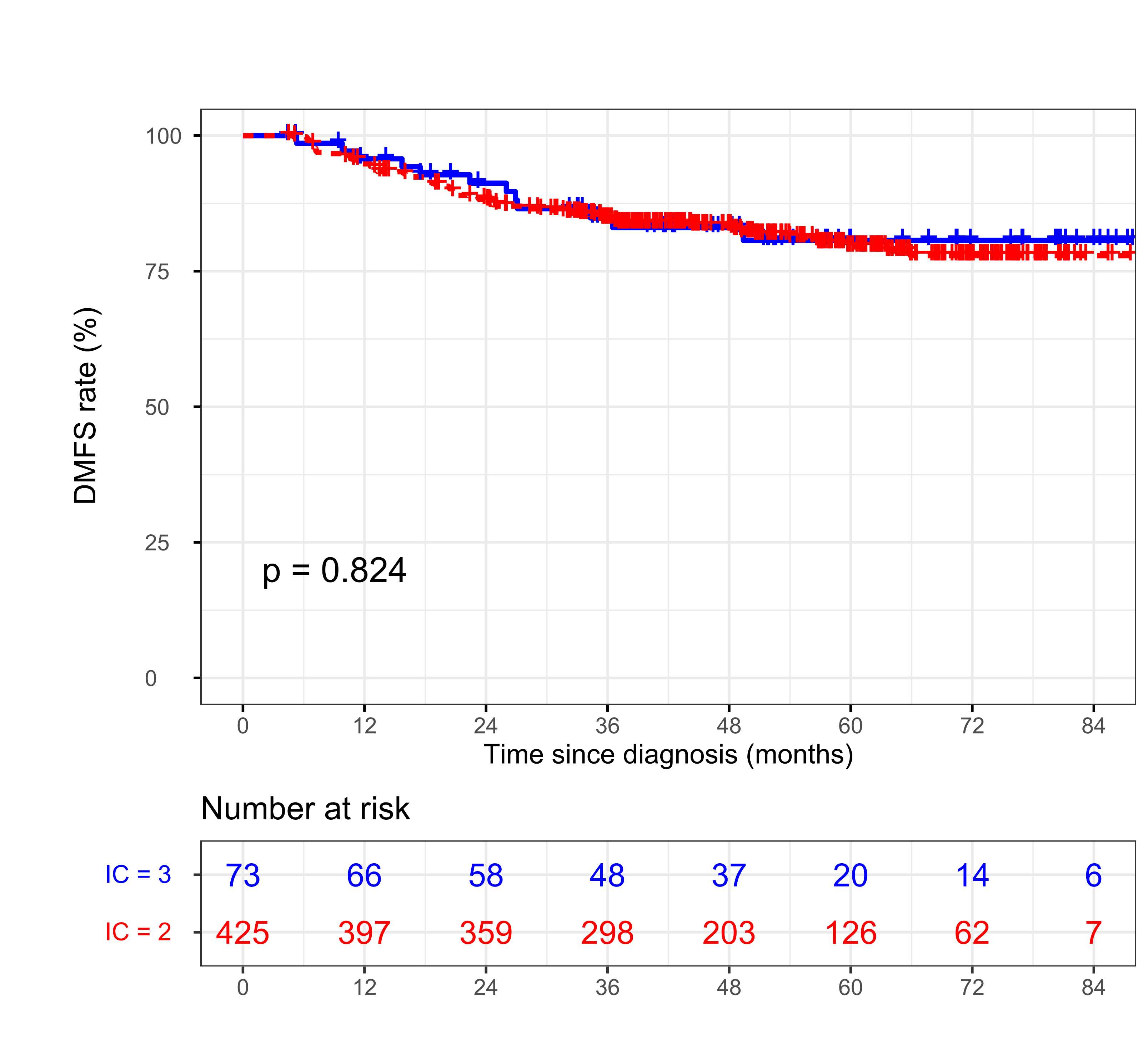

Supplement: Supplementary file 1 — Appendix S1 [file CAM4-12-4010-s001.zip › cam45256-sup-0001-AppendixS1/CAM4_5256_Figure S1C.Tiff]

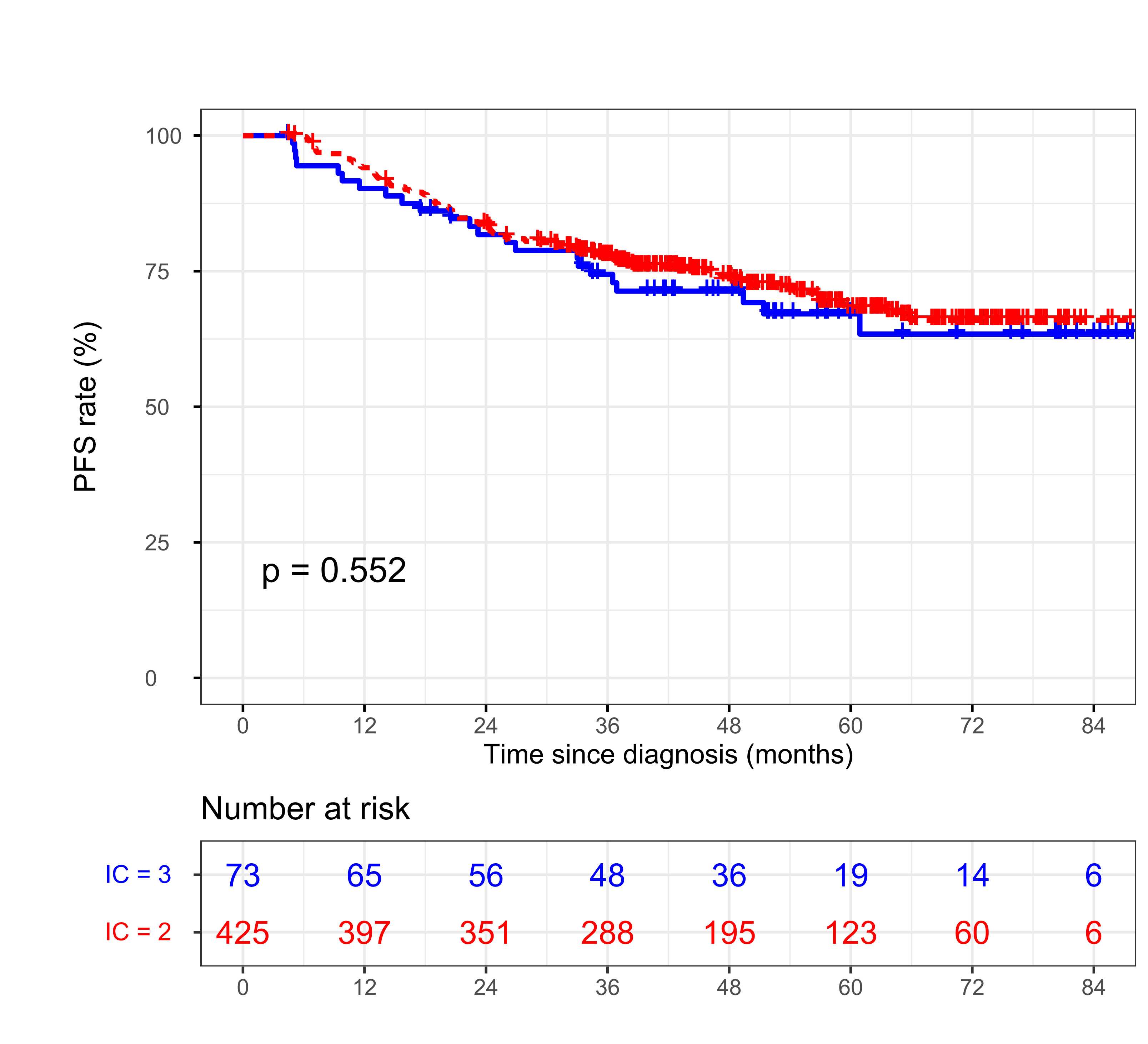

Supplement: Supplementary file 1 — Appendix S1 [file CAM4-12-4010-s001.zip › cam45256-sup-0001-AppendixS1/CAM4_5256_Figure S1D.Tiff]

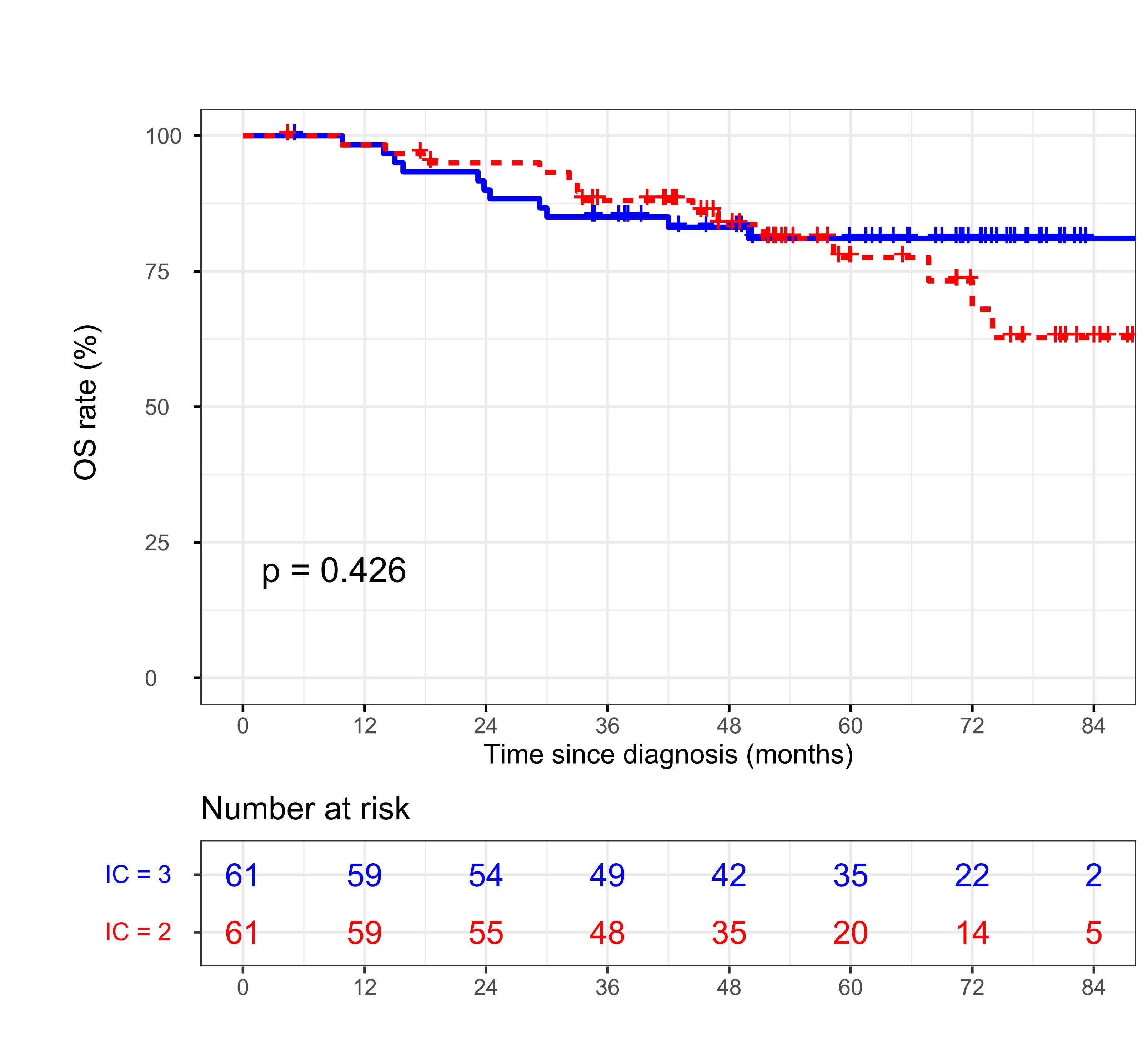

Supplement: Supplementary file 1 — Appendix S1 [file CAM4-12-4010-s001.zip › cam45256-sup-0001-AppendixS1/CAM4_5256_Figure S2A.Tiff]

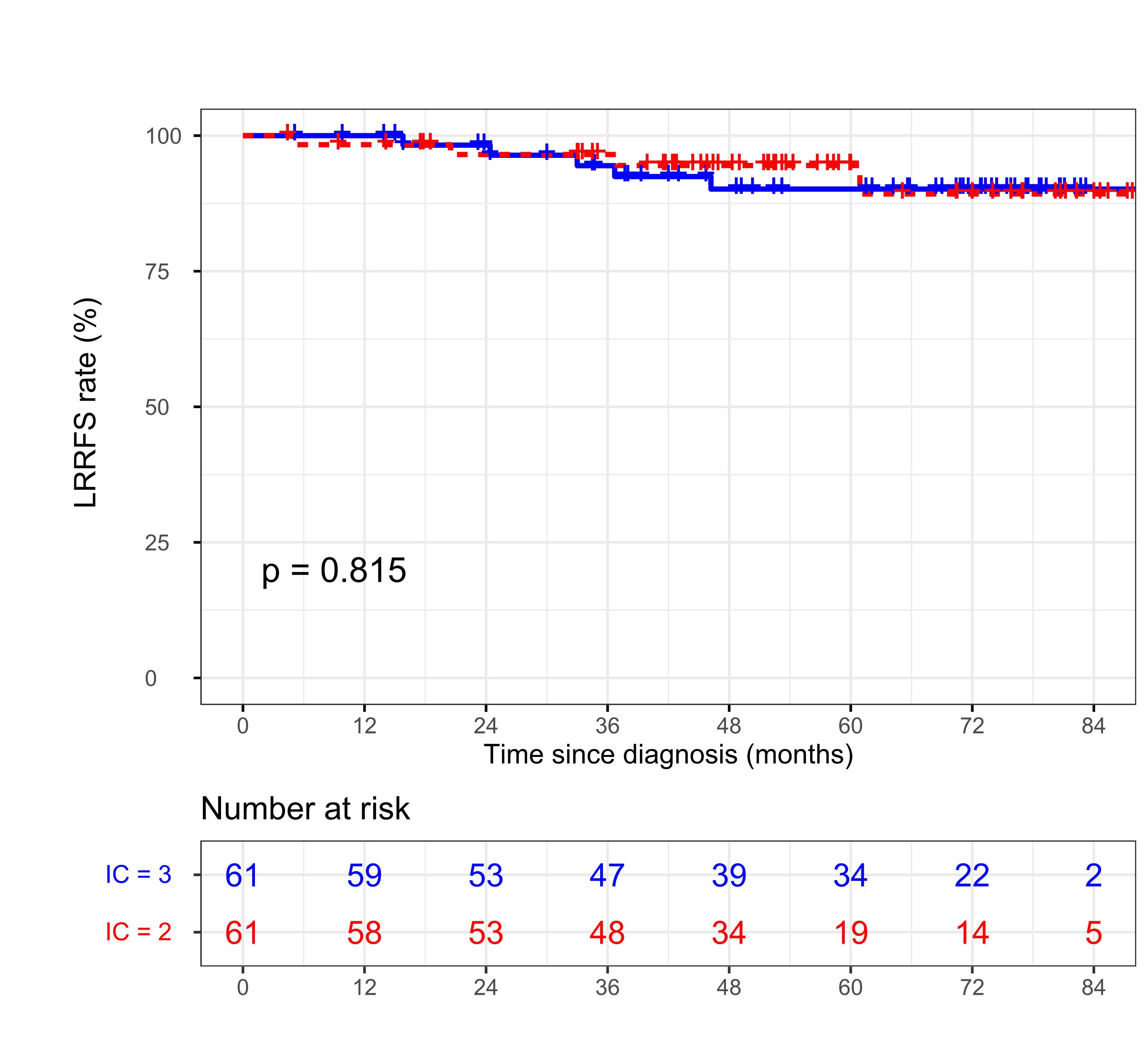

Supplement: Supplementary file 1 — Appendix S1 [file CAM4-12-4010-s001.zip › cam45256-sup-0001-AppendixS1/CAM4_5256_Figure S2B.Tiff]

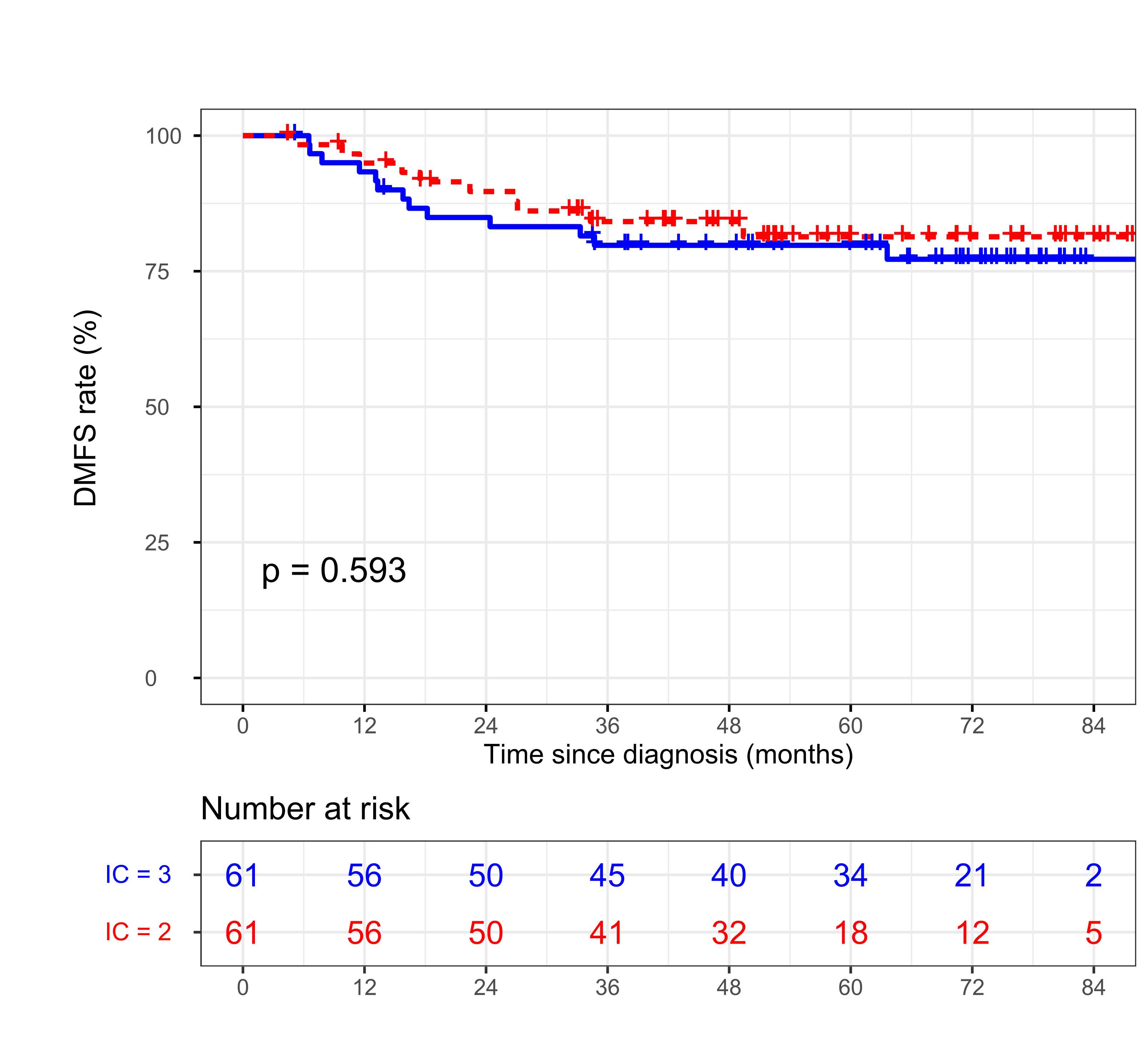

Supplement: Supplementary file 1 — Appendix S1 [file CAM4-12-4010-s001.zip › cam45256-sup-0001-AppendixS1/CAM4_5256_Figure S2C.Tiff]

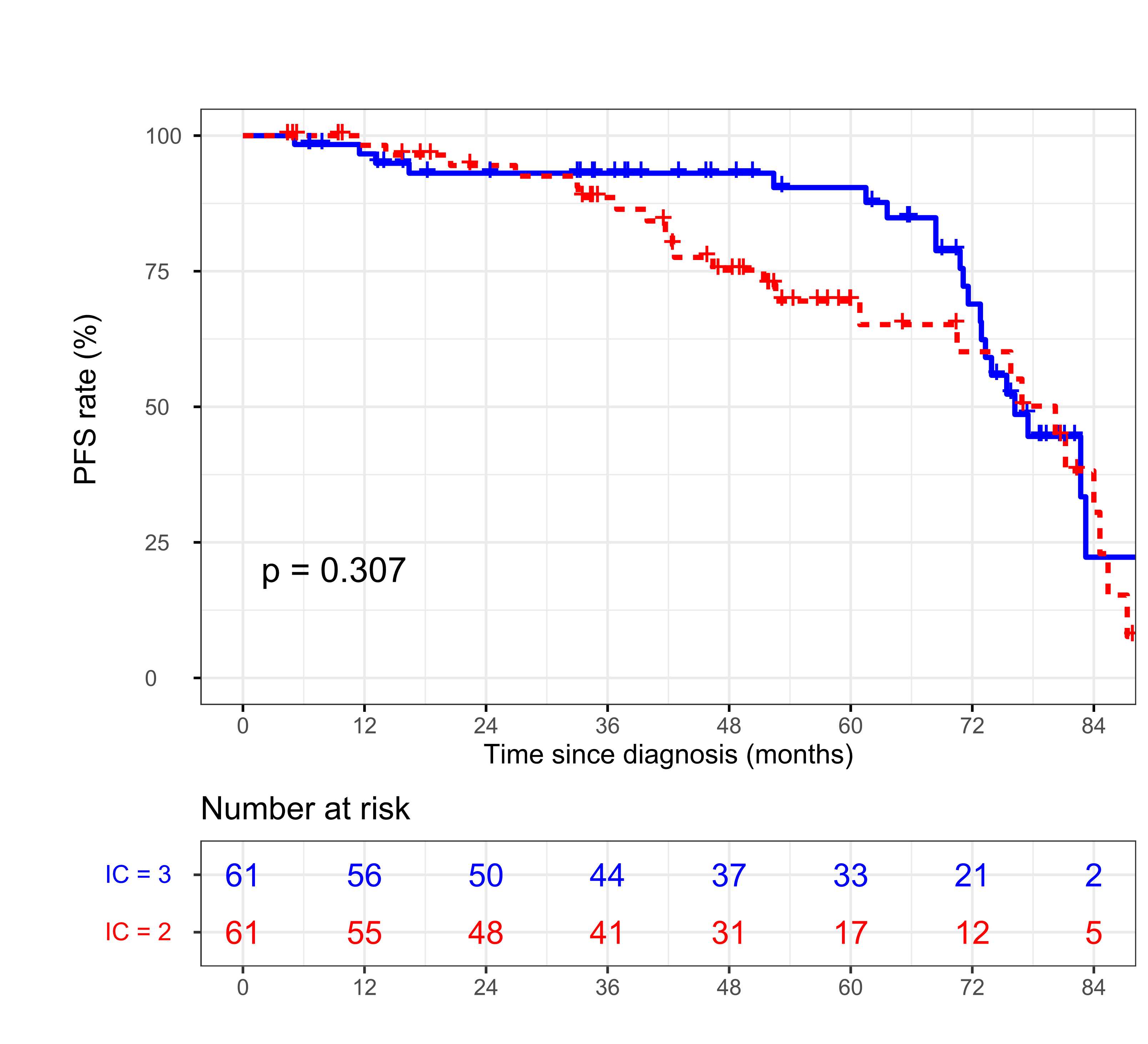

Supplement: Supplementary file 1 — Appendix S1 [file CAM4-12-4010-s001.zip › cam45256-sup-0001-AppendixS1/CAM4_5256_Figure S2D.Tiff]

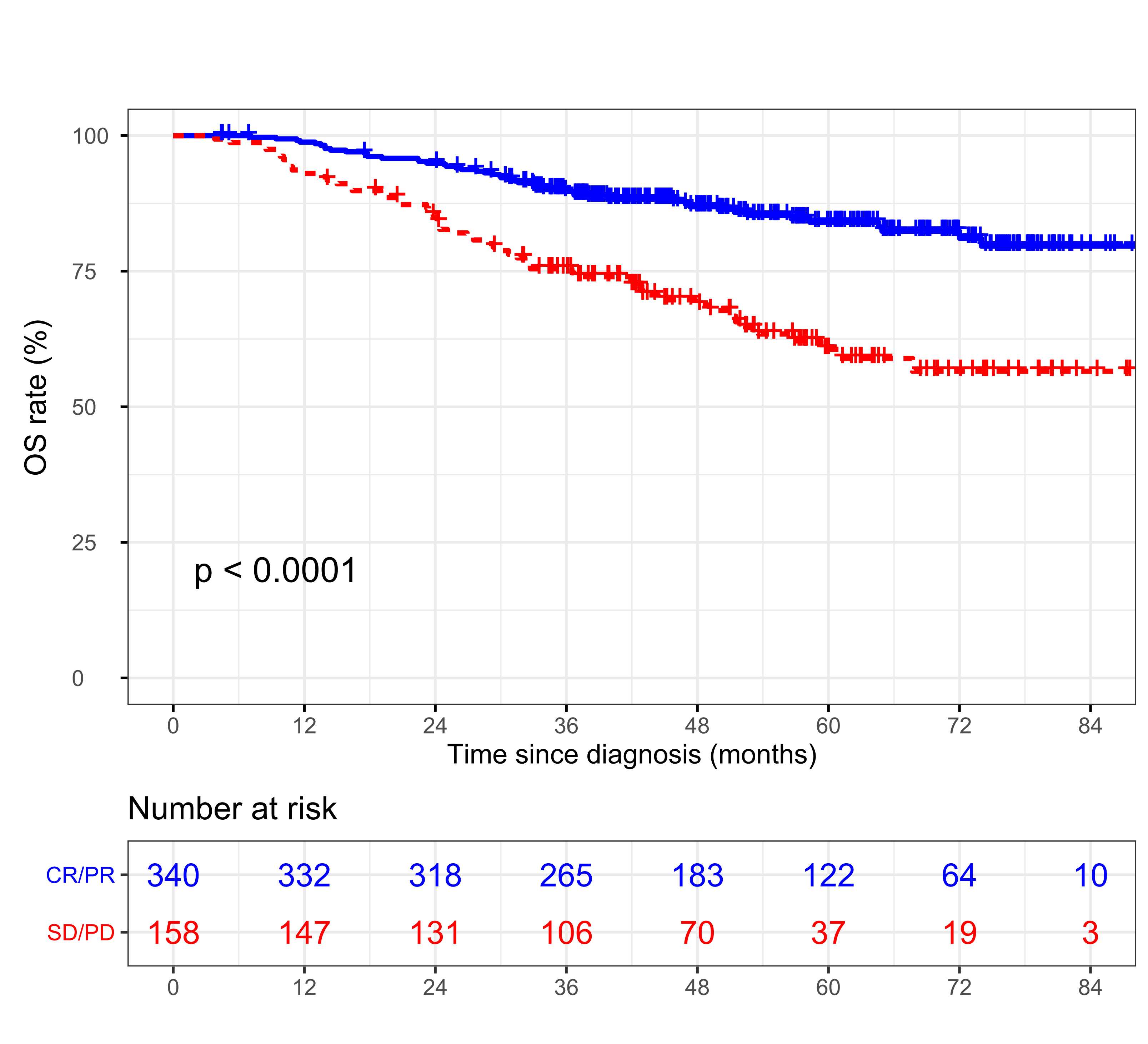

Supplement: Supplementary file 1 — Appendix S1 [file CAM4-12-4010-s001.zip › cam45256-sup-0001-AppendixS1/CAM4_5256_Figure S3A.Tiff]

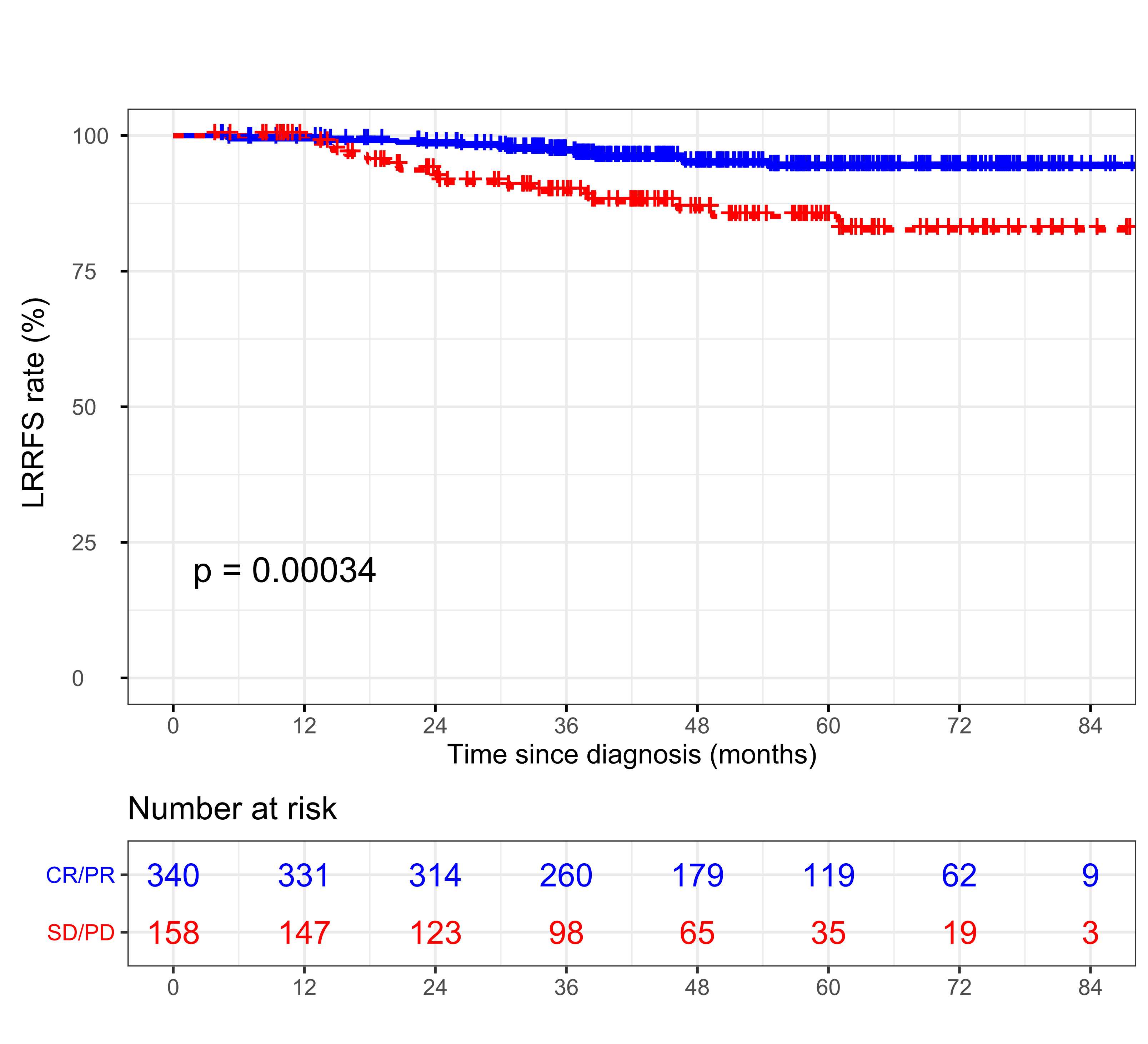

Supplement: Supplementary file 1 — Appendix S1 [file CAM4-12-4010-s001.zip › cam45256-sup-0001-AppendixS1/CAM4_5256_Figure S3B.Tiff]

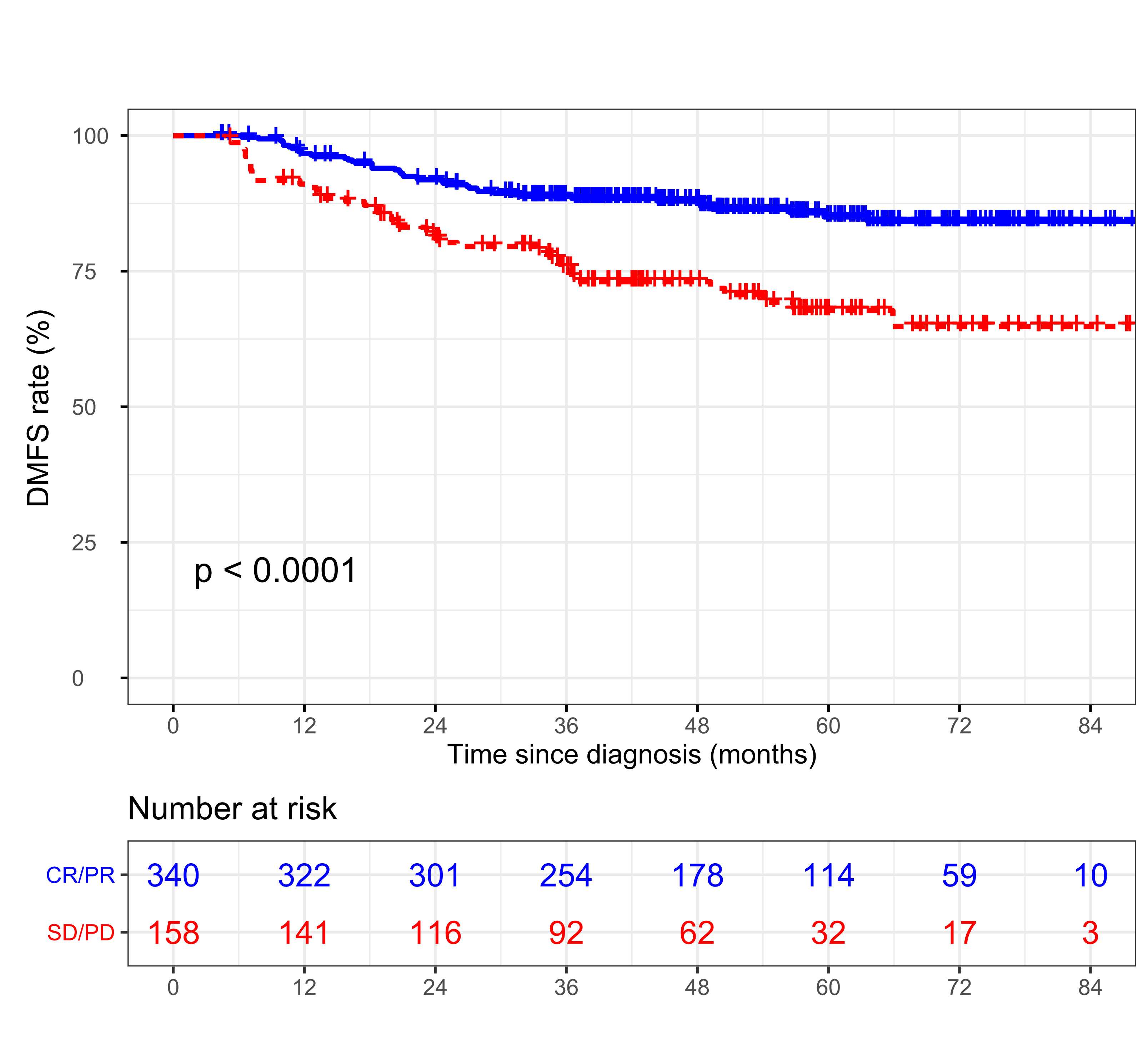

Supplement: Supplementary file 1 — Appendix S1 [file CAM4-12-4010-s001.zip › cam45256-sup-0001-AppendixS1/CAM4_5256_Figure S3C.Tiff]

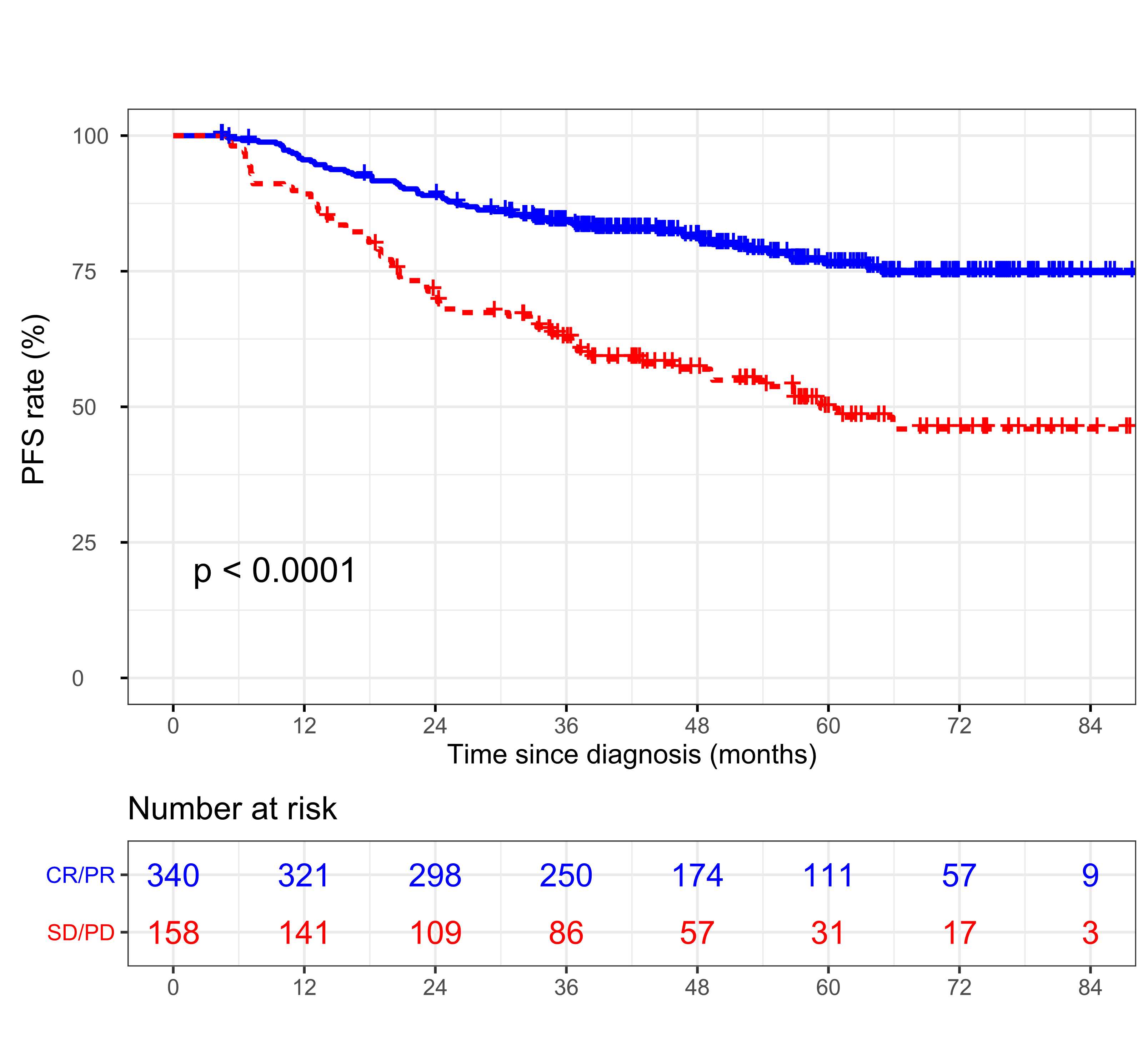

Supplement: Supplementary file 1 — Appendix S1 [file CAM4-12-4010-s001.zip › cam45256-sup-0001-AppendixS1/CAM4_5256_Figure S3D.Tiff]

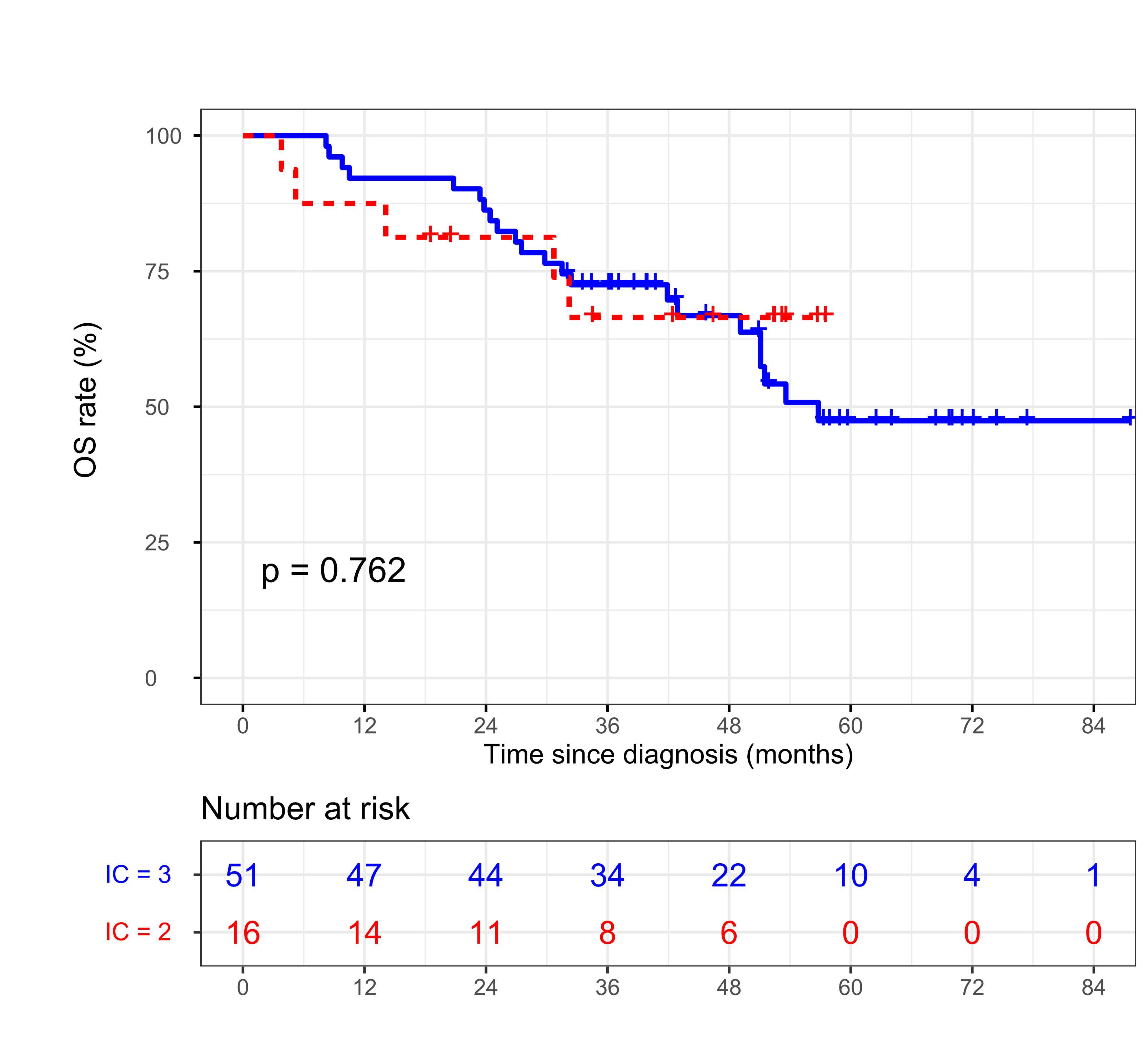

Supplement: Supplementary file 1 — Appendix S1 [file CAM4-12-4010-s001.zip › cam45256-sup-0001-AppendixS1/CAM4_5256_Figure S4A.Tiff]

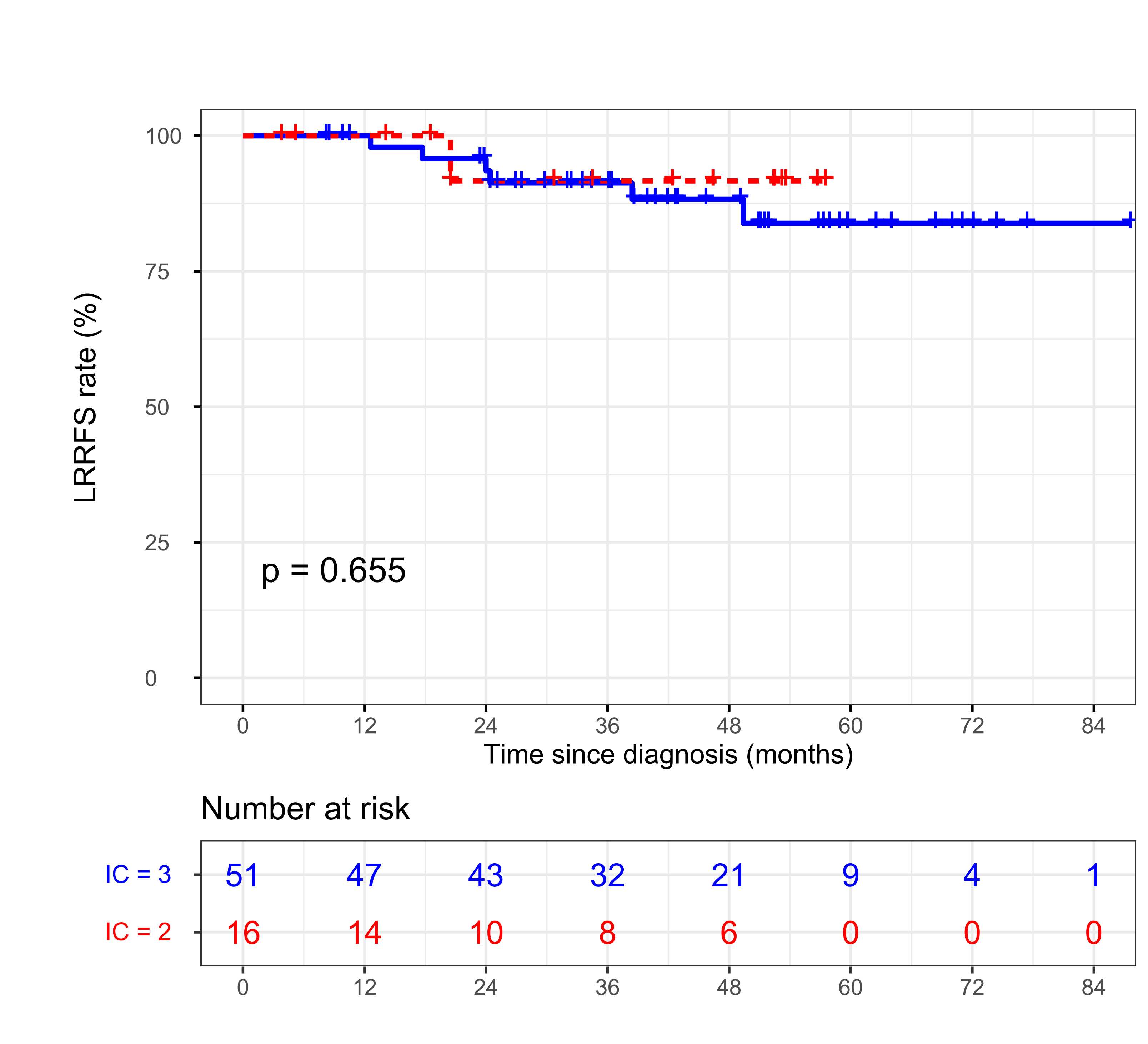

Supplement: Supplementary file 1 — Appendix S1 [file CAM4-12-4010-s001.zip › cam45256-sup-0001-AppendixS1/CAM4_5256_Figure S4B.Tiff]

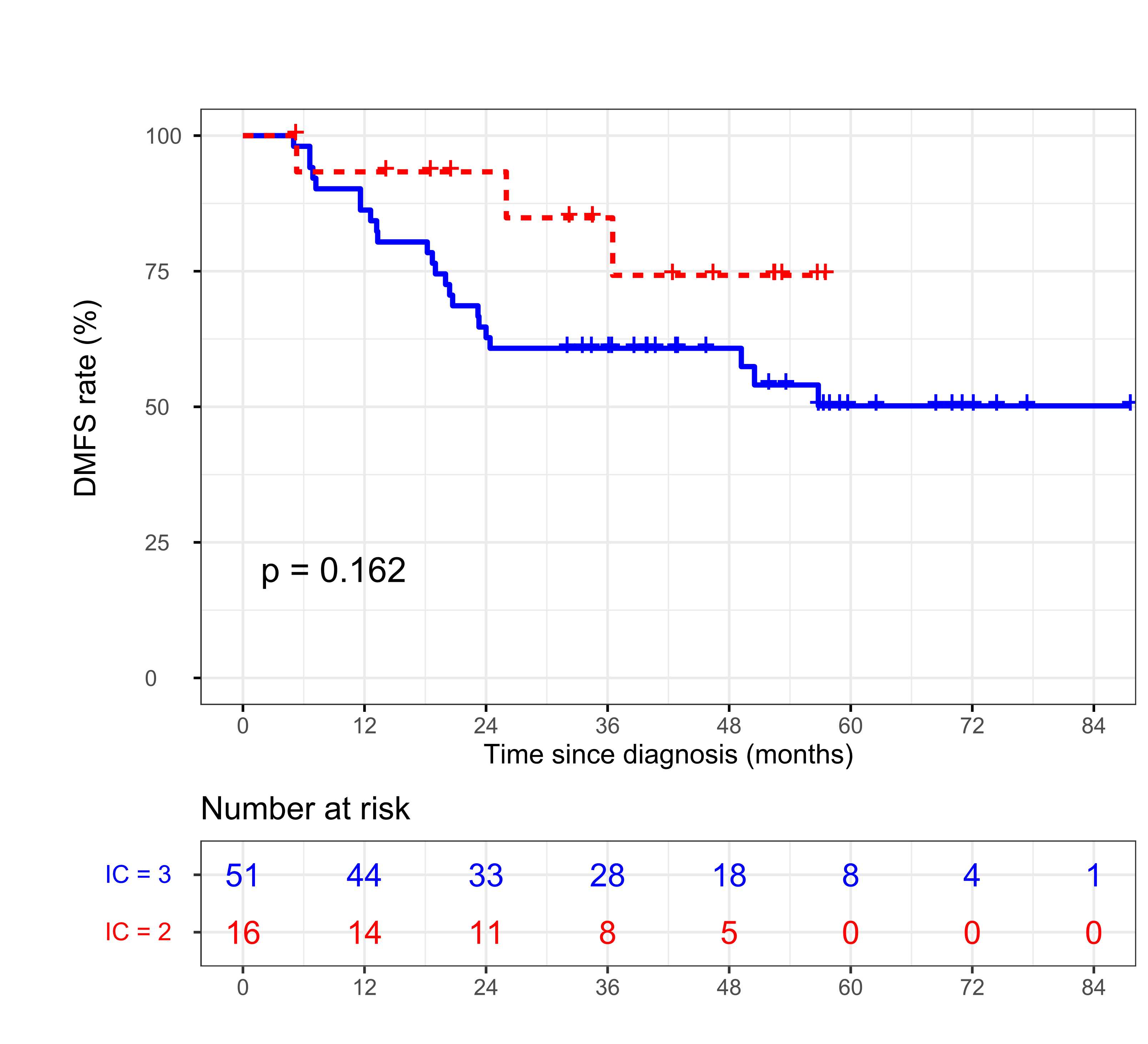

Supplement: Supplementary file 1 — Appendix S1 [file CAM4-12-4010-s001.zip › cam45256-sup-0001-AppendixS1/CAM4_5256_Figure S4C.Tiff]

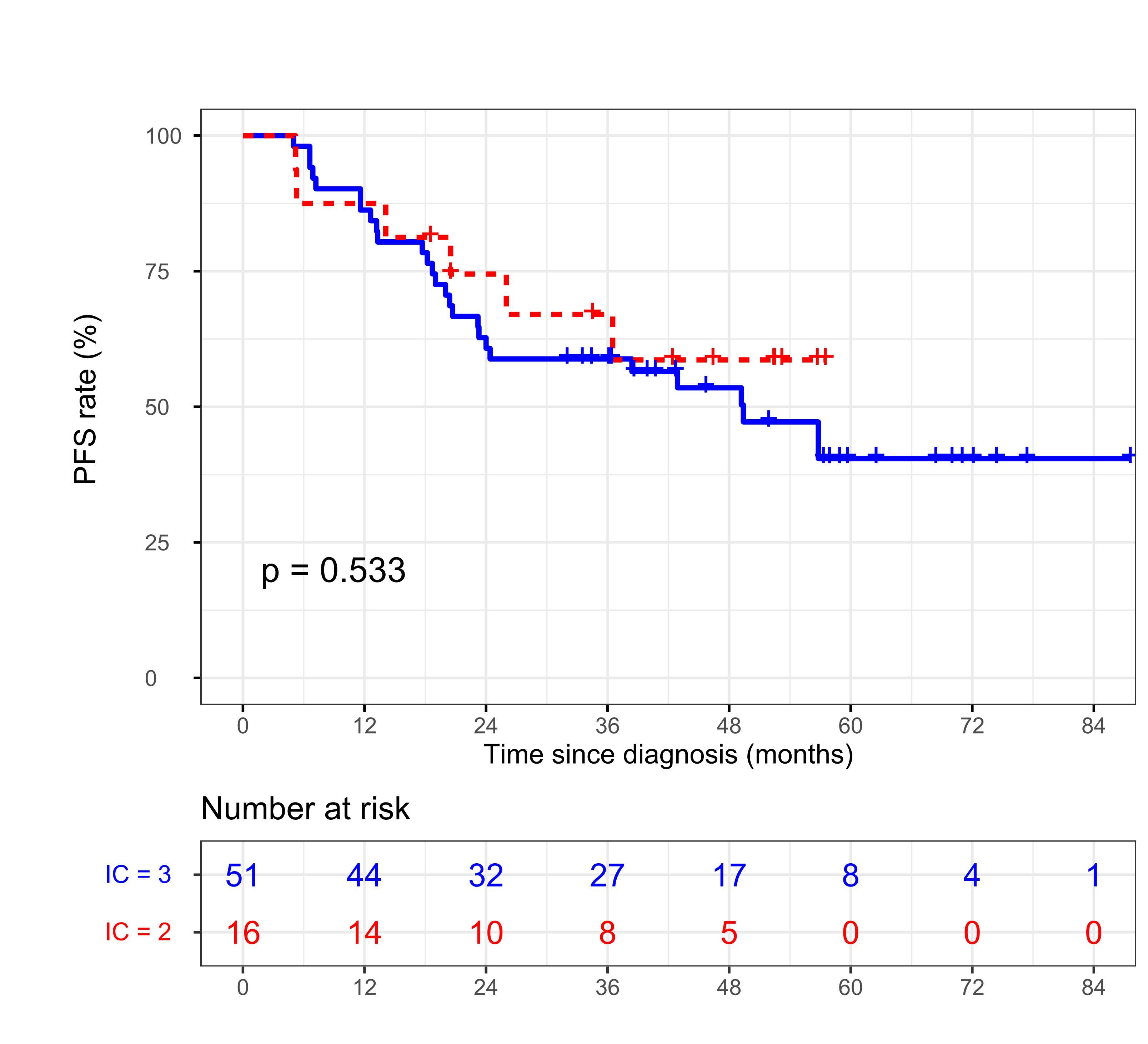

Supplement: Supplementary file 1 — Appendix S1 [file CAM4-12-4010-s001.zip › cam45256-sup-0001-AppendixS1/CAM4_5256_Figure S4D.Tiff]

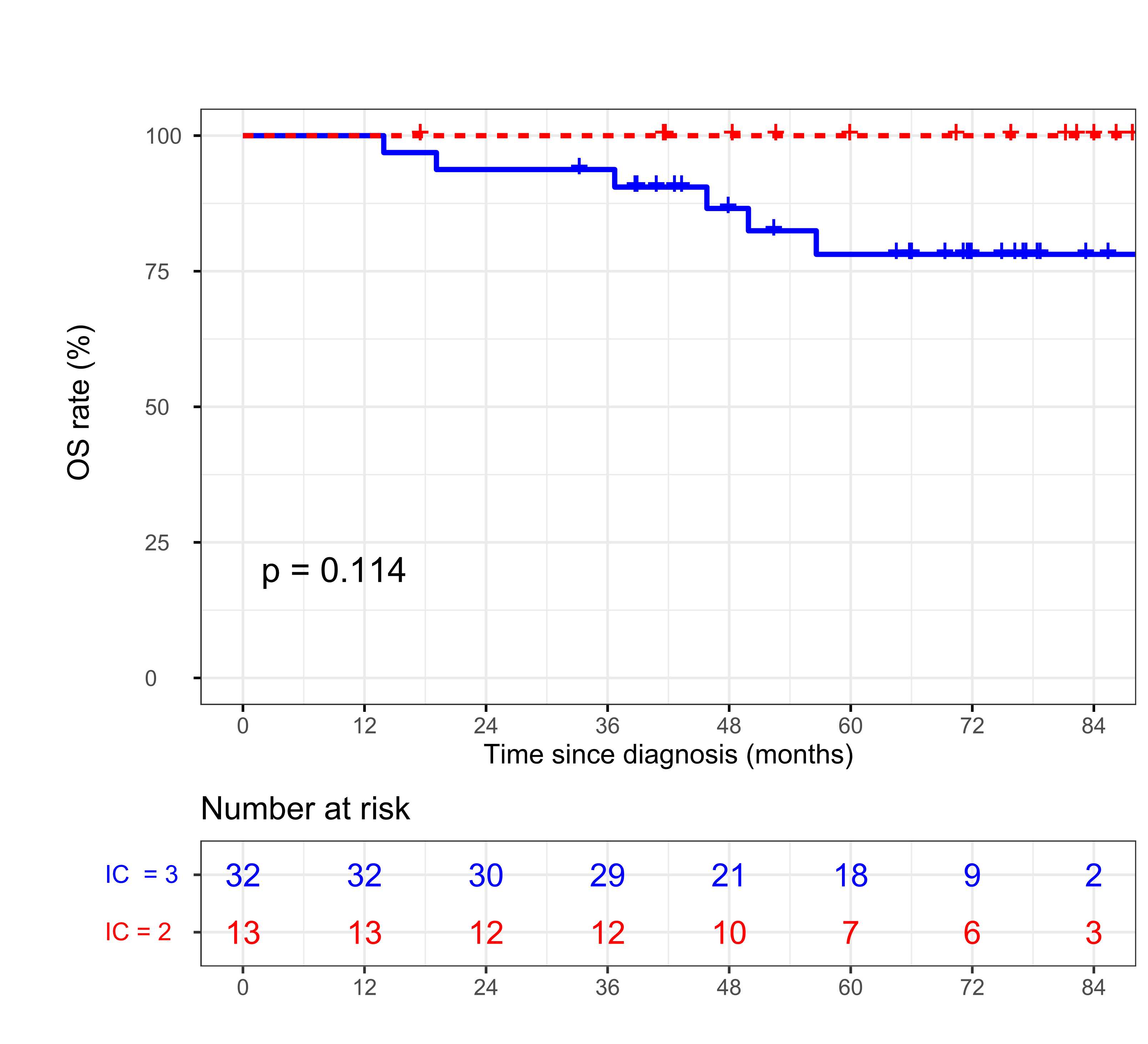

Supplement: Supplementary file 1 — Appendix S1 [file CAM4-12-4010-s001.zip › cam45256-sup-0001-AppendixS1/CAM4_5256_Figure S5A.Tiff]

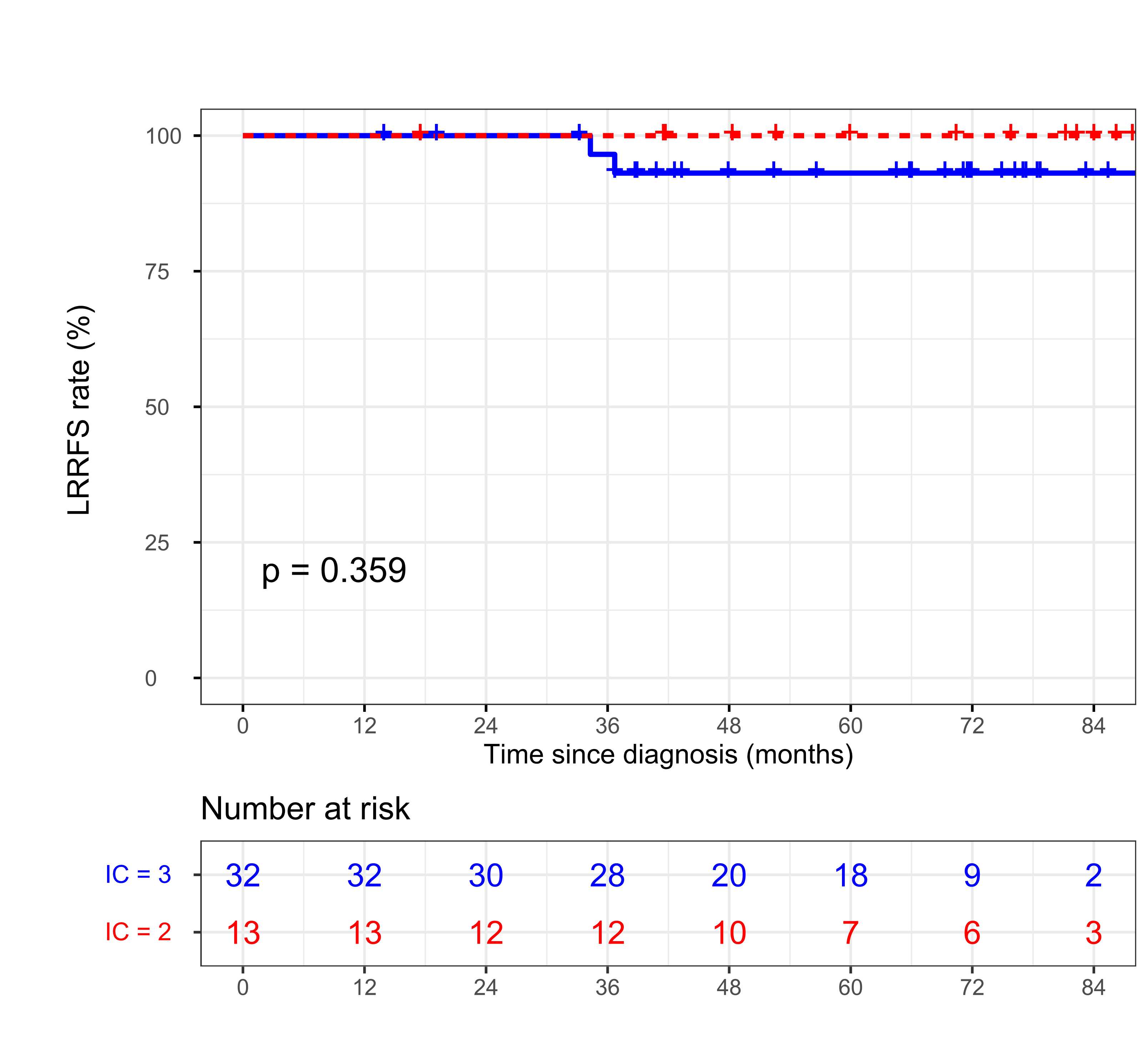

Supplement: Supplementary file 1 — Appendix S1 [file CAM4-12-4010-s001.zip › cam45256-sup-0001-AppendixS1/CAM4_5256_Figure S5B.Tiff]

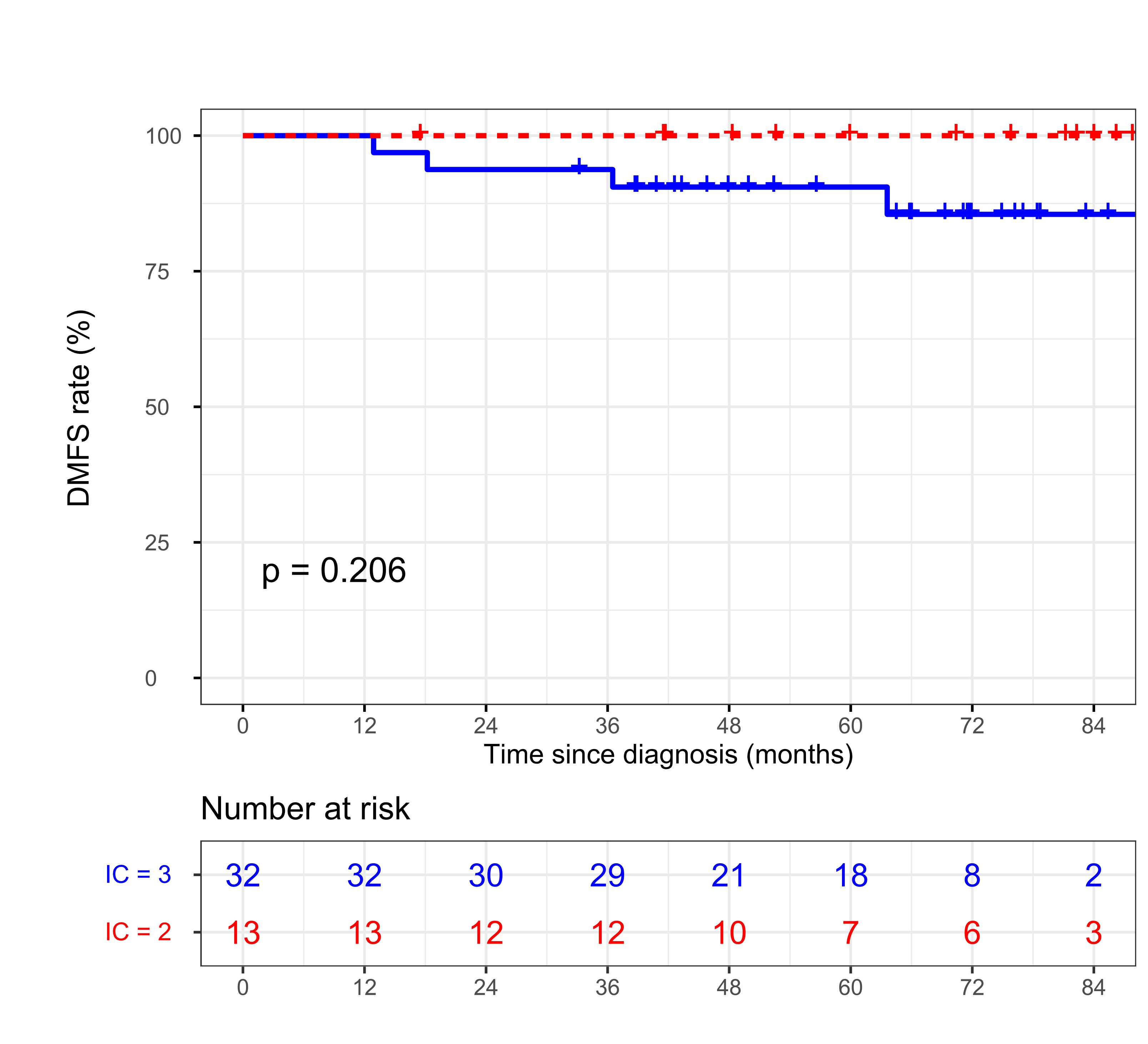

Supplement: Supplementary file 1 — Appendix S1 [file CAM4-12-4010-s001.zip › cam45256-sup-0001-AppendixS1/CAM4_5256_Figure S5C.Tiff]

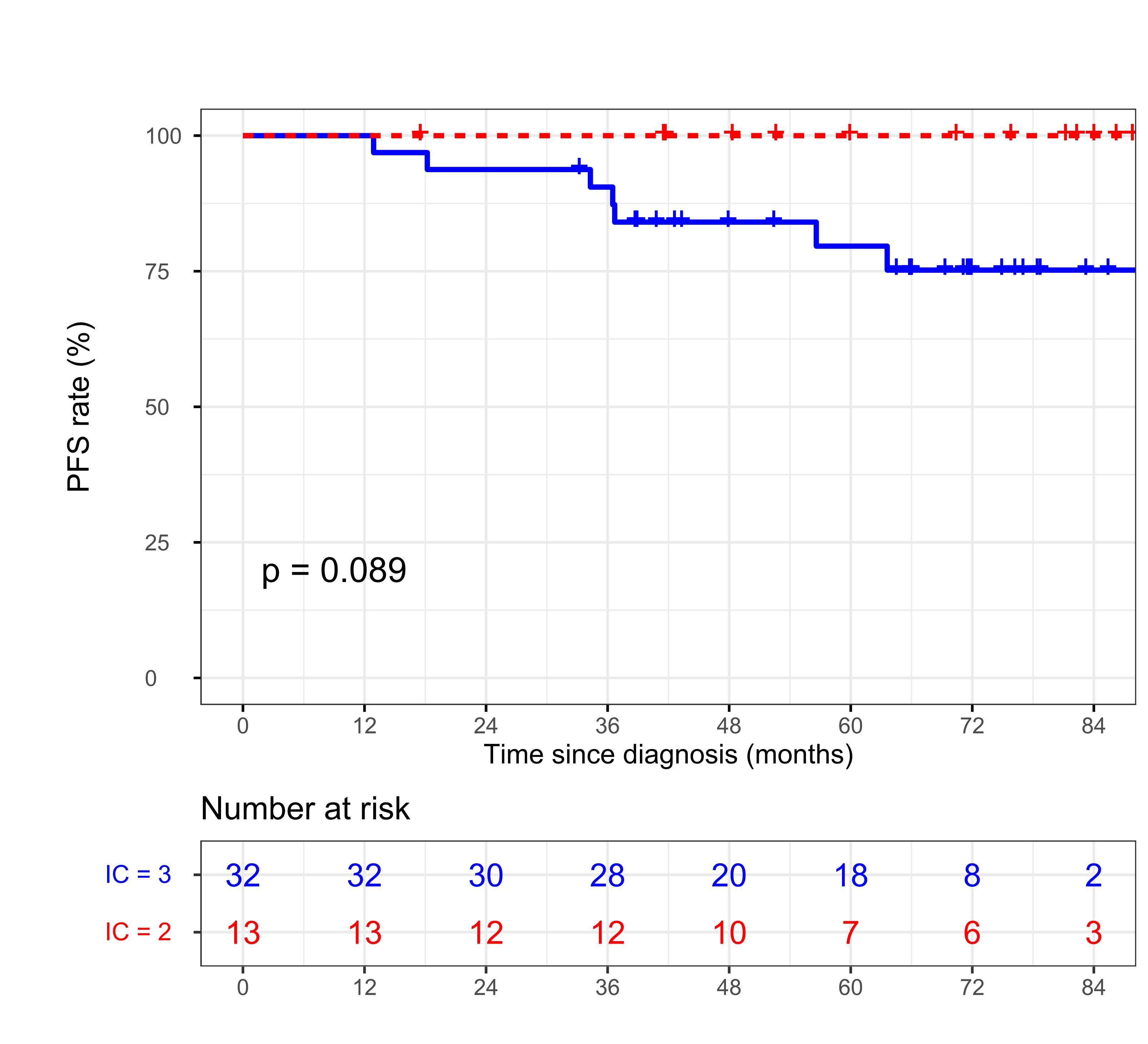

Supplement: Supplementary file 1 — Appendix S1 [file CAM4-12-4010-s001.zip › cam45256-sup-0001-AppendixS1/CAM4_5256_Figure S5D.Tiff]

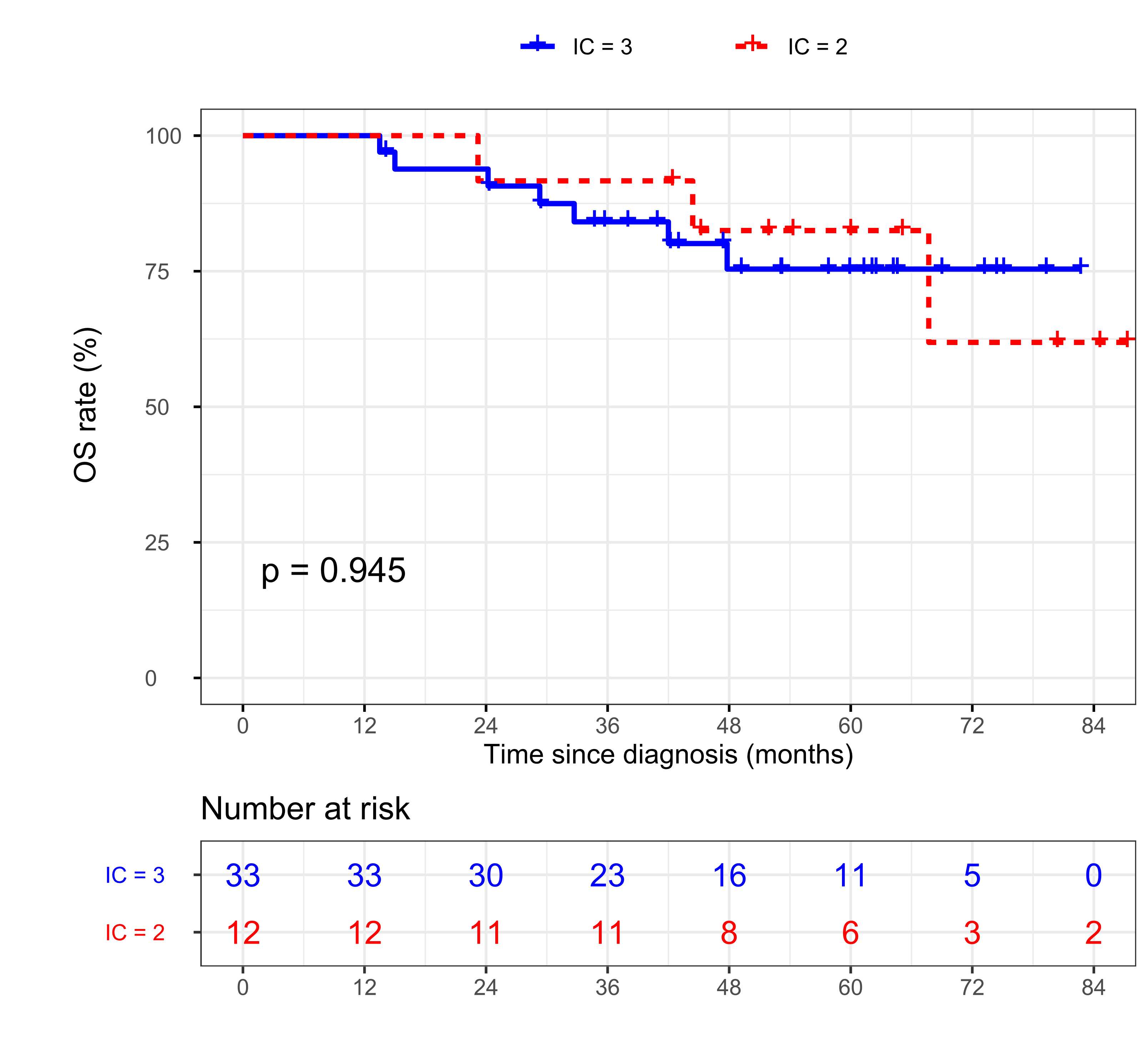

Supplement: Supplementary file 1 — Appendix S1 [file CAM4-12-4010-s001.zip › cam45256-sup-0001-AppendixS1/CAM4_5256_Figure S6A.Tiff]

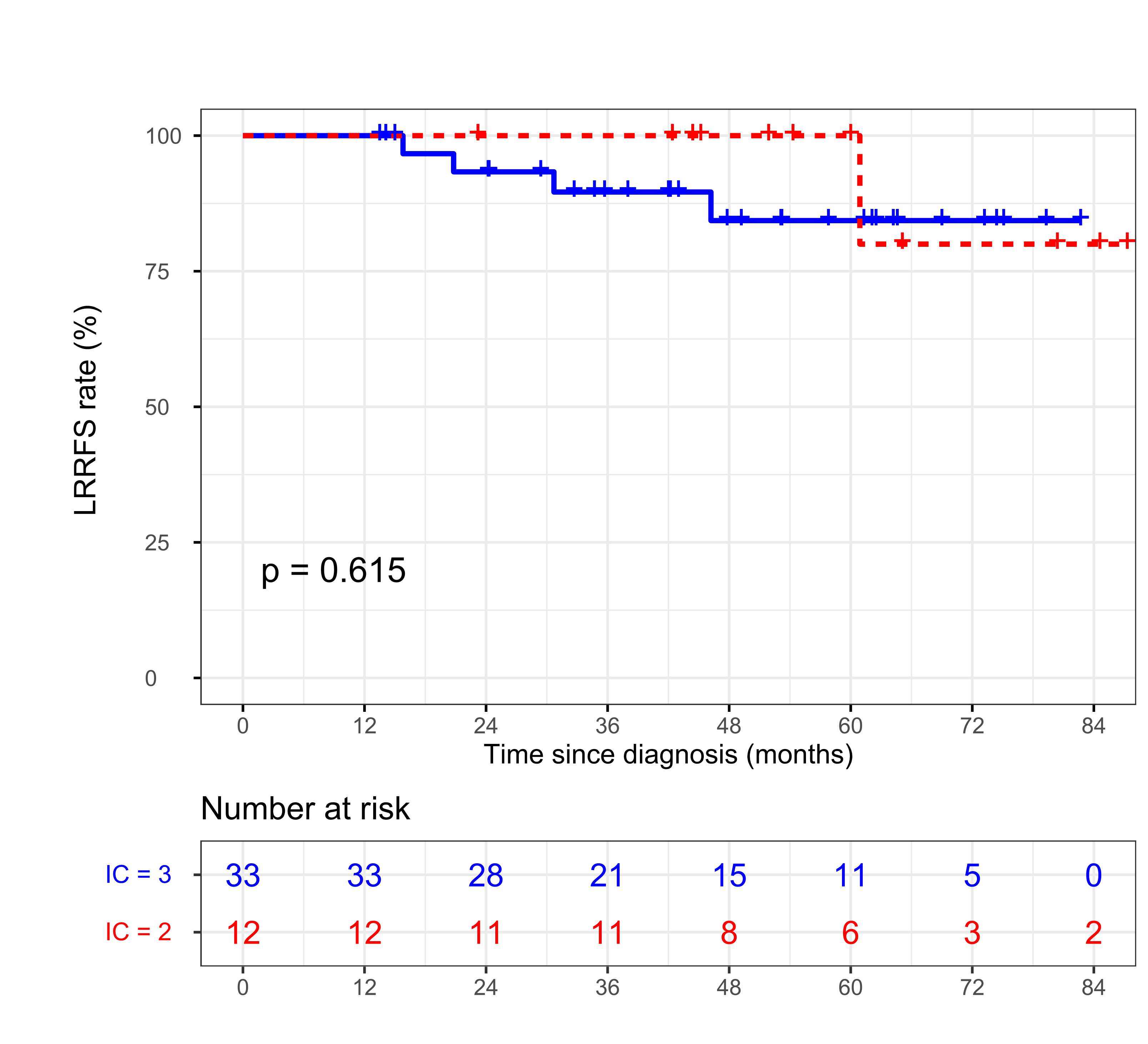

Supplement: Supplementary file 1 — Appendix S1 [file CAM4-12-4010-s001.zip › cam45256-sup-0001-AppendixS1/CAM4_5256_Figure S6B.Tiff]

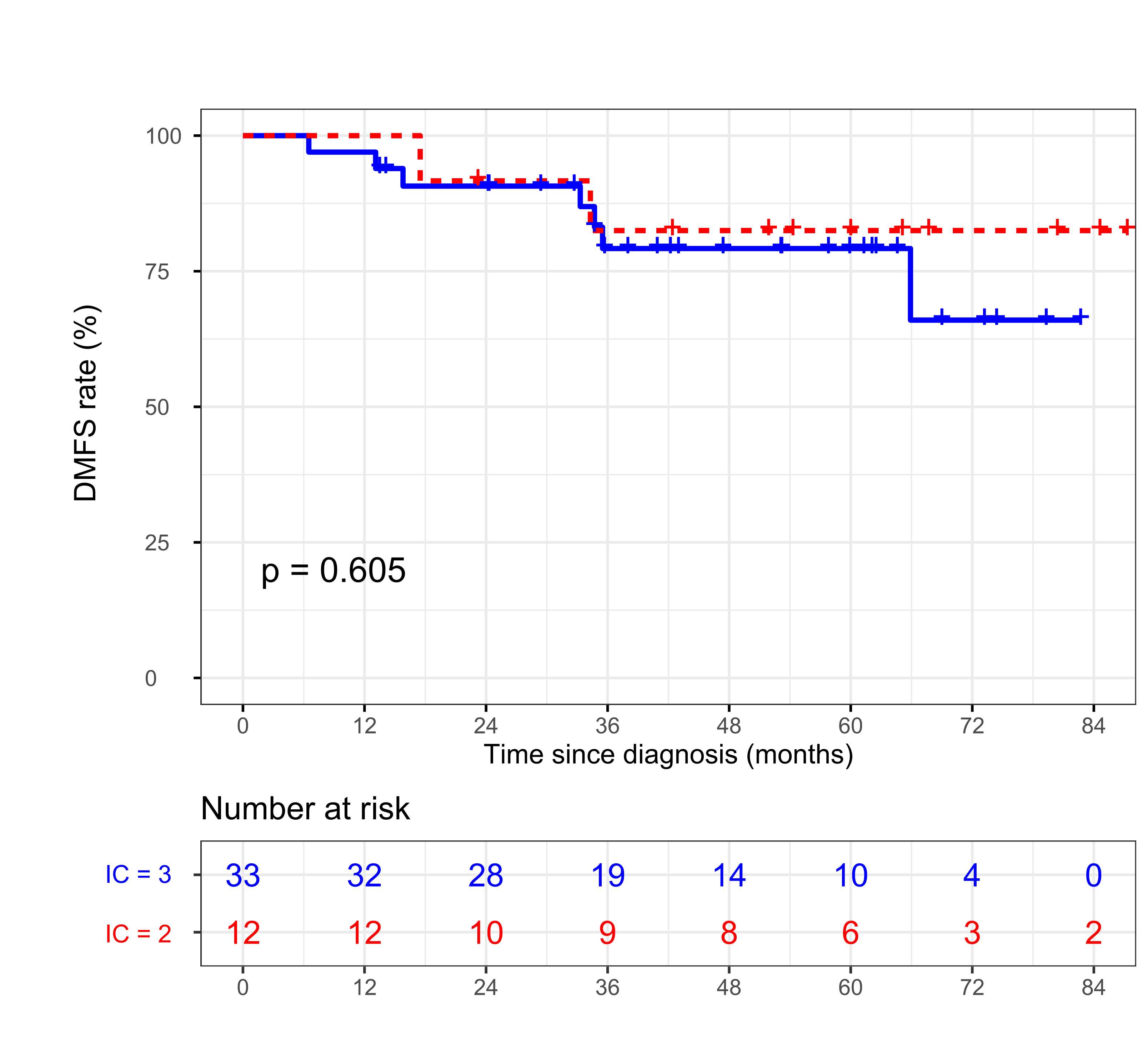

Supplement: Supplementary file 1 — Appendix S1 [file CAM4-12-4010-s001.zip › cam45256-sup-0001-AppendixS1/CAM4_5256_Figure S6C.Tiff]

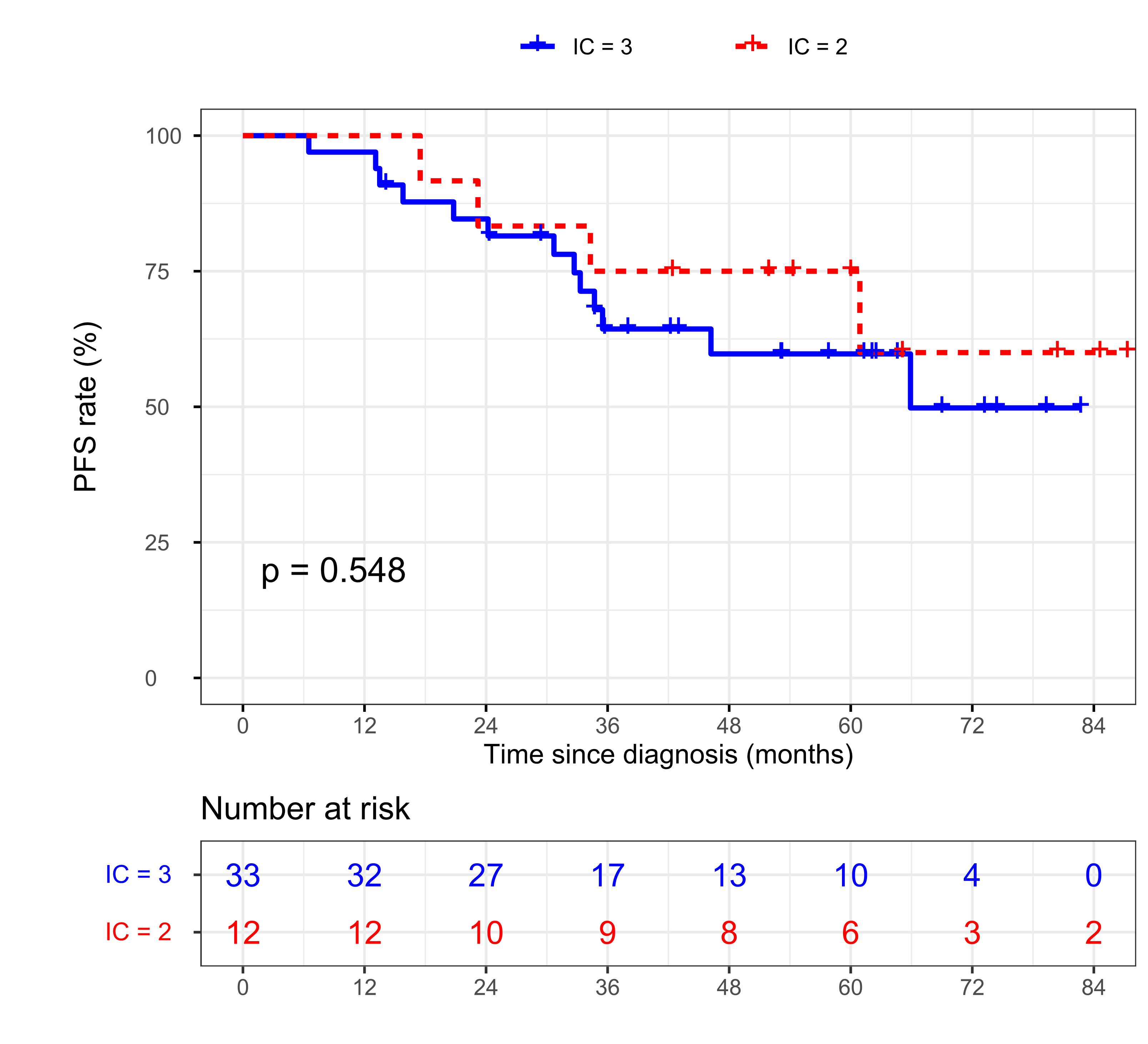

Supplement: Supplementary file 1 — Appendix S1 [file CAM4-12-4010-s001.zip › cam45256-sup-0001-AppendixS1/CAM4_5256_Figure S6D.Tiff]
