# Supplementary material for: Individualized number of induction chemotherapy cycles for locoregionally advanced nasopharyngeal carcinoma patients based on early tumor response
Source: Cancer Med. 2022 Sep 20;12(4):4010–22. doi: 10.1002/cam4.5256 (PMC9972137; doi:10.1002/cam4.5256)
Supplement: Supplementary file 2 — Appendix S2 [file CAM4-12-4010-s002.docx]

**Supplementary materials:**

**Details of treatment**

The regimens of IC included TPF (docetaxel 60 mg/m^2^ and cisplatin 60 mg/m^2^, day 1; fluorouracil 600 mg/m^2^/d, days 1-5), PF (cisplatin 80 mg/m^2^ day 1; fluorouracil 800 mg/m^2^/d, days 1-5), TP (docetaxel 75 mg/m^2^ and cisplatin 75 mg/m^2^, day 1), and GP (gemcitabine 1000 mg/m^2^, days 1 and 8; cisplatin 80 mg/m^2^, day 1) at three-week intervals. Concurrent chemotherapy is 3-weekly cisplatin (80–100 mg/m^2^) regimen for two to three cycles.

The primary gross tumor volume (GTVnx) and cervical lymph node tumor volume (GTVnd) included the entire macroscopic tumor defined with the aid of computed tomography (CT), magnetic resonance imaging (MRI) scans, and physical examinations. Two clinical target volumes (CTVs) were delineated according to the tumor invasion pattern. The high-risk clinical target volume (CTV1) included the GTVnx add a margin of 0.5 to 1 cm (forward, both sides, up and down) and a margin of 0.3 to 0.5 cm (back) to encompass the high-risk sites of microscopic extension and the whole nasopharynx. The low-risk clinical target volume (CTV2) was defined as the CTV1 add a margin of 0.5 to 1 cm (forward, both sides, up and down) and a margin of 0.3 to 0.5 cm (back) to encompass the low-risk sites of microscopic extension, the GTVnd, and elective neck area from level IB to V. The prescribed doses to the PTVs of GTVnx, GTVnd, CTV1, and CTV2 were 70.0~74.0 Gy/31~33 f, 60.0~73.6 Gy/30~33 f, 60.0~64.0 Gy/30~32 f, and 54.0~60.0 Gy/30~32 f, respectively.

**Supplementary Table 1** Basic characteristics of the patients in “CR/PR” and “SD/PD” groups stratified by IC cycle before PSM

| characteristic | CR/PR (n = 340) | | | SD/PD (n = 158) | | |
| --- | --- | --- | --- | --- | --- | --- |
|  | IC = 2 | IC = 3 | p-value | IC = 2 | IC = 3 | p-value |
| Total | 45 (13.2) | 295 (86.8) |  | 28 (17.7) | 130 (82.3) |  |
| Sex |  |  | 0.465 |  |  | 1.000 |
| Female | 14 (31.1) | 74 (25.1) |  | 7 (25.0) | 35 (26.9) |  |
| Male | 31 (68.9) | 221 (74.9) |  | 21 (75.0) | 95 (73.1) |  |
| Age (years) |  |  | 0.078 |  |  | 0.837 |
| ≤ 50 | 17 (37.8) | 155 (52.5) |  | 12 (42.9) | 59(45.4) |  |
| > 50 | 28 (62.2) | 140 (47.5) |  | 16 (57.1) | 71 (54.6) |  |
| Smoking status |  |  | 0.397 |  |  | 0.824 |
| No | 33 (73.3) | 195 (66.1) |  | 20 (71.4) | 88 (67.7) |  |
| Yes | 12 (26.7) | 100 (33.9) |  | 8 (28.6) | 42 (32.3) |  |
| Histology |  |  | 0.273 |  |  | 0.716 |
| WHO II | 6 (13.3) | 25 (8.5) |  | 3 (10.7) | 11 (8.5) |  |
| WHO III | 39 (86.7) | 270 (91.5) |  | 25 (89.3) | 119 (91.5) |  |
| T stage |  |  | 0.172 |  |  | 0.820 |
| T1 | 0 (0) | 8 (2.7) |  | 1 (3.6) | 3 (2.3) |  |
| T2 | 16 (35.6) | 72 (24.4) |  | 6 (21.4) | 22 (16.9) |  |
| T3 | 18 (40.0) | 106 (35.9) |  | 9 (32.1) | 37 (28.5) |  |
| T4 | 11 (24.4) | 109 (37.0) |  | 12 (42.9) | 68 (52.3) |  |
| N stage |  |  | 0.696 |  |  | 0.709 |
| N0 | 1 (2.2) | 6 (2.0) |  | 0 (0) | 3 (2.3) |  |
| N1 | 12 (26.7) | 76 (25.8) |  | 12 (42.9) | 51 (39.2) |  |
| N2 | 20 (44.4) | 109(36.9) |  | 10 (35.7) | 39 (30.0) |  |
| N3 | 12 (26.7) | 104 (35.3) |  | 6 (21.4) | 37 (28.5) |  |
| Overall stage (8th edition) |  |  | 0.046 |  |  | 0.064 |
| III | 23(51.1) | 103 (34.9) |  | 12 (42.9) | 32 (24.6) |  |
| IVA | 22(48.9) | 192 (65.1) |  | 16 (57.1) | 98 (75.4) |  |
| IC regimen |  |  | < 0.001 |  |  | 0.001 |
| TPF | 37 (82.2) | 235 (79.7) |  | 19 (69.7) | 113 (86.9) |  |
| TP | 3 (6.7) | 18 (6.1) |  | 4 (14.3) | 4 (3.1) |  |
| PF | 5 (11.1) | 3 (1.0) |  | 5 (17.9) | 4 (3.1) |  |
| GP | 0 (0) | 39 (13.2) |  | 0 (0) | 9 (6.9) |  |
| Concrrent chemotherapy cycles |  |  | 0.353 |  |  | 0.825 |
| 2 | 37 (82.2) | 222 (75.3) |  | 18 (64.3) | 88 (67.7) |  |
| 3 | 8 (17.8) | 73 (24.7) |  | 10 (35.7) | 42 (32.3) |  |

Note: Data are shown as number of patients (%).

Abbreviations: PSM, propensity score matching; CR, complete response; PR, partial response; SD, stable disease; PD, disease progression; WHO, World Health Organization; IC, induction chemotherapy; TPF, docetaxel-cisplatin-5-fluorouracil; TP, docetaxel-cisplatin; PF, cisplatin-5-fluorouracil; GP, gemcitabine-cisplatin.

**Supplementary Table 2** Five-year survival rates by IC cycle in different matched groups

| Endpoint | Events, n/N | | Survival rates, % | | HR (95% CI) | P value |
| --- | --- | --- | --- | --- | --- | --- |
|  | IC = 2 | IC = 3 | IC = 2 | IC = 3 |  |  |
| Overall survival |  |  |  |  |  |  |
| Unmatched entire cohort | 19/73 | 86/425 | 74.0 | 76.9 | 0.783 (0.476-1.288) | 0.334 |
| Matched entire cohort | 14/61 | 11/61 | 88.1 | 85.0 | 0.726 (0.329-1.604) | 0.426 |
| Matched CR/PR group | 11/45 | 14/90 | 75.4 | 83.7 | 0.604 (0.274-1.330) | 0.206 |
| Matched SD/PD group | 8/28 | 29/84 | 73.1 | 58.1 | 1.208 (0.552-2.645) | 0.635 |
| Matched CR/PR group with N0-1 stage | 0/13 | 6/32 | 100 | 73.8 | NA | 0.114 |
| Matched SD/PD group with N0-1 stage | 3/12 | 7/33 | 82.5 | 75.4 | 1.049 (0.270-4.078) | 0.945 |
| Matched CR/PR group with N2-3 stage | 11/32 | 8/58 | 64.6 | 87.5 | 0.361 (0.145-0.900) | 0.023 |
| Matched SD/PD group with N2-3 stage | 5/16 | 22/51 | 66.5 | 47.4 | 1.162 (0.439-3.077) | 0.762 |
| Locoregional relapse-free survival |  |  |  |  |  |  |
| Unmatched entire cohort | 5/73 | 29/425 | 93.9 | 91.1 | 0.994 (0.385-2.570) | 0.991 |
| Matched entire cohort | 4/61 | 5/61 | 90.1 | 94.5 | 1.170 (0.312-4.385) | 0.815 |
| Matched CR/PR group | 3/45 | 3/90 | 92.7 | 96.1 | 0.486 (0.098-2.409) | 0.367 |
| Matched SD/PD group | 2/28 | 10/84 | 95.8 | 83.9 | 1.660 (0.363-7.588) | 0.509 |
| Matched CR/PR group with N0-1 stage | 0/13 | 2/32 | 100 | 93.1 | NA | 0.359 |
| Matched SD/PD group with N0-1 stage | 1/12 | 14/33 | 100 | 84.3 | 1.742 (0.195-15.594) | 0.615 |
| Matched CR/PR group with N2-3 stage | 3/32 | 1/58 | 89.5 | 98.5 | 0.174 (0.018-1.671) | 0.086 |
| Matched SD/PD group with N2-3 stage | 1/16 | 6/51 | 91.7 | 83.8 | 1.613 (0.194-13.415) | 0.655 |
| Distant metastasis-free survival |  |  |  |  |  |  |
| Unmatched entire cohort | 12/73 | 77/425 | 80.7 | 79.5 | 1.071 (0.583-1.969) | 0.824 |
| Matched entire cohort | 10/61 | 13/61 | 79.8 | 81.3 | 1.252 (0.548-2.861) | 0.593 |
| Matched CR/PR group | 7/45 | 13/90 | 81.7 | 85.0 | 0.841 (0.335-2.109) | 0.711 |
| Matched SD/PD group | 5/28 | 30/84 | 78.5 | 61.4 | 2.065 (0.801-5.325) | 0.125 |
| Matched CR/PR group with N0-1 stage | 0/13 | 4/32 | 100 | 90.5 | NA | 0.206 |
| Matched SD/PD group with N0-1 stage | 2/12 | 7/33 | 82.5 | 79.2 | 1.512 (0.313-7.311) | 0.605 |
| Matched CR/PR group with N2-3 stage | 7/32 | 9/58 | 73.4 | 81.9 | 0.627 (0.233-1.686) | 0.350 |
| Matched SD/PD group with N2-3 stage | 3/16 | 23/51 | 74.2 | 50.2 | 2.304 (0.690-7.689) | 0.162 |
| Progression-free survival |  |  |  |  |  |  |
| Unmatched entire cohort | 23/73 | 121/425 | 67.1 | 68.0 | 0.873 (0.559-1.364) | 0.552 |
| Matched entire cohort | 24/61 | 22/61 | 69.5 | 90.4 | 0.734 (0.405-1.332) | 0.307 |
| Matched CR/PR group | 13/45 | 18/90 | 67.4 | 77.1 | 0.610 (0.299-1.245) | 0.170 |
| Matched SD/PD group | 10/28 | 40/84 | 66.1 | 48.1 | 1.395 (0.675-2.792) | 0.344 |
| Matched CR/PR group with N0-1 stage | 0/13 | 7/32 | 100 | 84.1 | NA | 0.312 |
| Matched SD/PD group with N0-1 stage | 4/12 | 13/33 | 75 | 59.0 | 1.409 (0.459-4.327) | 0.548 |
| Matched CR/PR group with N2-3 stage | 13/32 | 11/58 | 53.5 | 78.9 | 0.393 (0.176-0.879) | 0.018 |
| Matched SD/PD group with N2-3 stage | 6/16 | 27/51 | 58.7 | 40.5 | 1.324 (0.545-3.219) | 0.533 |

Abbreviations: CI, confidence interval, HR, hazard ratio; IC, induction chemotherapy; CR, complete response; PR, partial response; SD, stable disease; PD, disease progression.

**Supplementary Table 3** Univariable Cox analyses of prognostic factors in the whole cohort

| characteristic | HR(95%CI) | p-value |
| --- | --- | --- |
| OS |  |  |
| Sex (male vs. female) | 1.205 (0.764-1.901) | 0.422 |
| Age (> 50 vs. ≤ 50) | 1.885 (1.263-2.814) | 0.002 |
| Smoking status (yes vs. no) | 1.185 (0.796-1.765) | 0.403 |
| Histology (WHO III vs. WHO I/II) | 0.846 (0.463-1.544 | 0.586 |
| T stage (T3/4 vs. T1/2) | 0.698 (0.462-1.055) | 0.088 |
| N stage (N2/3 vs. N0/1) | 1.588 (1.024-2.462) | 0.039 |
| Overall stage (IVA vs. II/III) | 2.027 (1.304-3.291) | 0.002 |
| IC regimen (TPF vs. others) | 1.495 (0.835-2.677) | 0.176 |
| IC cycles (3 vs. 2) | 0.783 (0.476-1.094) | 0.336 |
| Tumor response (SD/PD vs. CR/PR) | 2.734 (1.863-4.012) | < 0.001 |
| Concrrent chemotherapy cycles (3 vs. 2) | 0.984 (0.635-1.526) | 0.944 |
| LRRFS |  |  |
| Sex (male vs. female) | 0.843 (0.403-1.762) | 0.649 |
| Age (> 50 vs. ≤ 50) | 1.458 (0.737-2.888) | 0.279 |
| Smoking status (yes vs. no) | 1.297 (0.649-2.591) | 0.461 |
| Histology (WHO III vs. WHO I/II) | 1.716 (0.411-7.164) | 0.459 |
| T stage (T3/4 vs. T1/2) | 0.743 (0.254-2.175) | 0.588 |
| N stage (N2/3 vs. N0/1) | 1.137 (0.554-2.335) | 0.727 |
| Overall stage (IVA vs. II/III) | 0.940 (0.471-1.878) | 0.861 |
| IC regimen (TPF vs. others) | 2.301 (0.702-7.573) | 0.169 |
| IC cycles (3 vs. 2) | 0.994 (0.385-2.570) | 0.991 |
| Tumor response (SD/PD vs. CR/PR) | 3.226 (1.638-6.352) | 0.001 |
| Concrrent chemotherapy cycles (3 vs. 2) | 0.851 (0.385-1.880) | 0.690 |
| DMFS |  |  |
| Sex (male vs. female) | 1.320 (0.795-2.192) | 0.283 |
| Age (> 50 vs. ≤ 50) | 0.933 (0.616-1.414) | 0.743 |
| Smoking status (yes vs. no) | 1.170 (0.759-1.804) | 0.477 |
| Histology (WHO III vs. WHO I/II) | 0.826 (0.428-1.596) | 0.596 |
| T stage (T3/4 vs. T1/2) | 0.648 (0.417-1.005) | 0.053 |
| N stage (N2/3 vs. N0/1) | 1.995 (1.200-3.315) | 0.008 |
| Overall stage (IVA vs. II/III) | 1.891 (1.159-3.086)- | 0.011 |
| IC regimen (TPF vs. others) | 1.442 (0.784-2.652) | 0.240 |
| IC cycles (3 vs. 2) | 1.071 (0.583-1.969) | 0.824 |
| Tumor response (SD/PD vs. CR/PR) | 2.450 (1.616-3.715) | < 0.001 |
| Concrrent chemotherapy cycles (3 vs. 2) | 1.106 (0.696-1.756) | 0.670 |
| PFS |  |  |
| Sex (male vs. female) | 1.072 (0.735-1.564) | 0.717 |
| Age (> 50 vs. ≤ 50) | 1.288 (0.926-1.790) | 0.132 |
| Smoking status (yes vs. no) | 1.121 (0.795-1.580) | 0.515 |
| Histology (WHO III vs. WHO I/II) | 1.084 (0.613-1.919) | 0.781 |
| T stage (T3/4 vs. T1/2) | 0.795 (0.555-1.139) | 0.211 |
| N stage (N2/3 vs. N0/1) | 1.445 (1.002-2.082) | 0.049 |
| Overall stage (IVA vs. II/III) | 1.779 (1.219-2.594) | 0.003 |
| IC regimen (TPF vs. others) | 1.307 (0.822-2.079) | 0.258 |
| IC cycles (3 vs. 2) | 0.873 (0.559-1.364) | 0.552 |
| Tumor response (SD/PD vs. CR/PR) | 2.684 (1.935-3.724) | < 0.001 |
| Concrrent chemotherapy cycles (3 vs. 2) | 1.304 (0.895-1.898) | 0.167 |

Abbreviations: WHO, World Health Organization; IC, induction chemotherapy; TPF, docetaxel-cisplatin-5-fluorouracil; Others, included TP, GP, PF regimens; CR, complete response; PR, partial response; SD, stable disease; PD, disease progression; OS, overall survival; LRRFS, locoregional relapse-free survival; DMFS, distant metastasis-free survival; PFS, progression-free survival; CI, confidence interval, HR, hazard ratio.

**Supplementary Table 4** Multivariable analysis of prognostic factors in unmatched “CR/PR” and “SD/PD” cohorts

| characteristic | CR/PR (n = 340) |  | SD/PD (n = 158) |  |
| --- | --- | --- | --- | --- |
|  | HR (95%CI) | p-value | HR (95%CI) | p-value |
| OS |  |  |  |  |
| Age (> 50 vs. ≤ 50) | 1.435 (0.801-2.572) | 0.225 | 2.121 (1.172-3.841) | 0.013 |
| T stage (T3/4 vs. T1/2) | 0.852 (0.451-1.610) | 0.621 | 0.832 (0.426-1.623) | 0.589 |
| N stage (N2/3 vs. N0/1) | 1.513 (0.727-3.149) | 0.275 | 1.889 (0.998-3.575) | 0.051 |
| IC cycles (3 vs. 2) | 0.615 (0.313-1.211) | 0.160 | 1.195 (0.561-2.546) | 0.645 |
| IC regimen (TPF vs. others) | 1.325 (0.558-3.147) | 0.523 | 1.675 (0.753-3.724) | 0.206 |
| Concrrent chemotherapy cycles (3 vs. 2) | 0.721 (0.333-1.562) | 0.407 | 1.299 (0.740-2.281) | 0.362 |
| LRRFS |  |  |  |  |
| Age (> 50 vs. ≤ 50) | 1.870 (0.621-5.630) | 0.266 | 0.921 (0.353-2.398) | 0.865 |
| T stage (T3/4 vs. T1/2) | 0.935 (0.282-3.098) | 0.912 | 0.597 (0.181-1.976) | 0.597 |
| N stage (N2/3 vs. N0/1) | 1.211 (0.340-4.310) | 0.767 | 1.239 (0.434-3.540) | 0.689 |
| IC cycles (3 vs. 2) | 0.715 (0.200-2.564) | 0.607 | 1.917 (0.440-8.354) | 0.386 |
| IC regimen (TPF vs. others) | 2.642 (0.343-20.331) | 0.351 | 2.018 (0.463-8.802) | 0.350 |
| Concrrent chemotherapy cycles (3 vs. 2) | 0.601 (0.133-2.730) | 0.510 | 1.007 (0.375-2.705) | 0.989 |
| DMFS |  |  |  |  |
| Age (> 50 vs. ≤ 50) | 0.755 (0.413-1.382) | 0.363 | 0.904 (0.486-1.679) | 0.749 |
| T stage (T3/4 vs. T1/2) | 1.028 (0.528-2.000) | 0.936 | 0.506 (0.247-1.033) | 0.061 |
| N stage (N2/3 vs. N0/1) | 2.071 (0.918-4.672) | 0.079 | 1.949 (0.918-4.136) | 0.082 |
| IC cycles (3 vs. 2) | 0.779 (0.344-1.766) | 0.550 | 1.741 (0.682-4.444) | 0.246 |
| IC regimen (TPF vs. others) | 1.401 (0.587-3.347) | 0.448 | 1.306 (0.548-3.113) | 0.546 |
| Concrrent chemotherapy cycles (3 vs. 2) | 0.546 (0.241-1.240) | 0.148 | 1.626 (0.885-2.990) | 0.117 |
| PFS |  |  |  |  |
| Age (> 50 vs. ≤ 50) | 1.010 (0.625-1.632) | 0.968 | 1.307 (0.801-2.133) | 0.283 |
| T stage (T3/4 vs. T1/2) | 0.975 (0.570-1.667) | 0.925 | 0.824 (0.449-1.510) | 0.531 |
| N stage (N2/3 vs. N0/1) | 1.723 (0.929-3.193) | 0.084 | 1.559 (0.914-2.658) | 0.103 |
| IC cycles (3 vs. 2) | 0.665 (0.361-1.225) | 0.191 | 1.403 (0.716-2.751) | 0.324 |
| IC regimen (TPF vs. others) | 1.219 (0.618-2.402) | 0.568 | 1.314 (0.689-2.509) | 0.407 |
| Concrrent chemotherapy cycles (3 vs. 2) | 0.532 (0.269-1.051) | 0.069 | 1.167 (0.711-1.918) | 0.541 |

Abbreviations: IC, induction chemotherapy; CR, complete response; PR, partial response; SD, stable disease; PD, disease progression; OS, overall survival; LRRFS, locoregional relapse-free survival; DMFS, distant metastasis-free survival; PFS, progression-free survival; CI, confidence interval, HR, hazard ratio.

**Supplementary Table 5** Basic characteristics of the N2-3 patients in the matched “CR/PR” and “SD/PD” cohorts stratified by IC cycle

| characteristic | CR/PR (n = 90) | | | SD/PD (n = 67) | | |
| --- | --- | --- | --- | --- | --- | --- |
|  | IC = 2 | IC = 3 | p-value | IC = 2 | IC = 3 | p-value |
| Total | 32 | 58 |  | 16 | 51 |  |
| Sex |  |  | 0.631 |  |  | 0.182 |
| Female | 8 (25.0) | 18 (31.0) |  | 6 (37.5) | 10 (19.6) |  |
| Male | 24 (75.0) | 40 (69.0) |  | 10 (62.5) | 41 (80.4) |  |
| Age (years) |  |  | 1.000 |  |  | 0.553 |
| ≤ 50 | 11 (34.4) | 20 (34.5) |  | 7 (43.8) | 17 (33.3) |  |
| > 50 | 21 (65.6) | 38 (65.5) |  | 9 (56.3) | 34 (66.7) |  |
| Smoking status |  |  | 1.000 |  |  | 0.760 |
| No | 24 (75.0) | 43 (74.1) |  | 11 (68.8) | 37 (72.5) |  |
| Yes | 8 (25.0) | 15 (25.9) |  | 5 (31.3) | 14 (27.5) |  |
| Histology |  |  | 1.000 |  |  | 0.623 |
| WHO II | 5 (15.6) | 9 (15.5) |  | 2 (12.5) | 4 (7.8) |  |
| WHO III | 27 (84.4) | 49 (84.5) |  | 14 (87.5) | 47 (92.2) |  |
| T stage |  |  | 0.852 |  |  | 0.821 |
| T1 | 0 (0) | 1 (1.7) |  | 1 (6.3) | 2 (3.9) |  |
| T2 | 16 (50.0) | 28 (48.3) |  | 6 (37.5) | 19 (37.3) |  |
| T3 | 10 (31.3) | 16 (27.6) |  | 6 (37.5) | 15 (29.4) |  |
| T4 | 6 (18.8) | 13 (22.4) |  | 3 (18.8) | 15 (29.4) |  |
| N stage |  |  | 1.000 |  |  | 0.400 |
| N0 | 0 (0) | 0 (0) |  | 0 (0) | 0 (0) |  |
| N1 | 0 (0) | 0 (0) |  | 0 (0) | 0 (0) |  |
| N2 | 20 (62.5) | 36 (62.1) |  | 10 (62.5) | 25 (49.0) |  |
| N3 | 12 (37.5) | 22 (37.9) |  | 6 (37.5) | 26 (51.0) |  |
| Overall stage (8th edition) |  |  | 1.000 |  |  | 0.085 |
| III | 15 (46.9) | 28 (48.3) |  | 9 (56.3) | 16 (31.4) |  |
| IVA | 17 (53.1) | 30 (51.7) |  | 7 (43.8) | 35 (68.6) |  |
| IC regimen |  |  | 0.488 |  |  | 0.447 |
| TPF | 27 (84.4) | 50 (86.2) |  | 12 (75.0) | 40 (78.4) |  |
| TP | 2 (6.3) | 4 (6.9) |  | 3 (18.8) | 4 (7.8) |  |
| PF | 3 (9.4) | 2 (3.4) |  | 1 (6.3) | 3 (5.9) |  |
| GP | 0 (0) | 2 (3.4) |  | 0 (0) | 4 (7.8) |  |
| Concrrent chemotherapy cycles |  |  | 0.521 |  |  | 0.204 |
| 2 | 27 (84.4) | 50 (86.2) |  | 11 (68.8) | 34 (66.7) |  |
| 3 | 5 (15.6) | 8 (13.8) |  | 5 (31.3) | 17 (33.3) |  |

Note: Data are shown as number of patients (%).

Abbreviations: CR, complete response; PR, partial response; SD, stable disease; PD, disease progression; WHO, World Health Organization; IC, induction chemotherapy; TPF, docetaxel-cisplatin-5-fluorouracil; TP, docetaxel-cisplatin; PF, cisplatin-5-fluorouracil; GP, gemcitabine-cisplatin.

**Supplementary Table 6** Basic characteristics of the N0-1 patients in matched “CR/PR” and “SD/PD” cohorts stratified by IC cycle

| characteristic | CR/PR (n = 45) | | | SD/PD (n = 45) | | |
| --- | --- | --- | --- | --- | --- | --- |
|  | IC = 2 | IC = 3 | p-value | IC = 2 | IC = 3 | p-value |
| Total | 13 | 32 |  | 12 | 33 |  |
| Sex |  |  | 0.743 |  |  | 0.655 |
| Female | 6 (46.2) | 18 (56.3) |  | 1 (8.3) | 6 (18.2) |  |
| Male | 7 (53.8) | 14 (43.8) |  | 11 (91.7) | 27 (81.8) |  |
| Age (years) |  |  | 0.751 |  |  | 0.501 |
| ≤ 50 | 6 (46.2) | 13 (40.6) |  | 5 (41.7) | 19 (57.6) |  |
| > 50 | 7 (53.8) | 19 (59.4) |  | 7 (58.3) | 14 (42.4) |  |
| Smoking status |  |  | 0.441 |  |  | 1.000 |
| No | 9 (69.2) | 26 (81.3) |  | 9 (75.0) | 26 (78.8) |  |
| Yes | 4 (30.8) | 6 (18.8) |  | 3 (25.5) | 7 (21.2) |  |
| Histology |  |  | 0.405 |  |  | 1.000 |
| WHO II | 1 (7.7) | 7 (21.9) |  | 1 (8.3) | 4 (12.1) |  |
| WHO III | 12 (92.3) | 25 (78.1) |  | 11 (91.7) | 29 (87.9) |  |
| T stage |  |  | 0.094 |  |  | 0.488 |
| T1 | 0 (0) | 0 (0) |  | 0 (0) | 0 (0) |  |
| T2 | 0 (0) | 0 (0) |  | 0 (0) | 0 (0) |  |
| T3 | 8 (61.5) | 10 (31.3) |  | 3 (25.0) | 14 (42.4) |  |
| T4 | 5 (38.5) | 22 (68.8) |  | 9 (75.0) | 19 (57.6) |  |
| N stage |  |  | 1.000 |  |  | 1.000 |
| N0 | 1 (7.7) | 3 (9.4) |  | 0 (0) | 1 (3.0) |  |
| N1 | 12 (92.3) | 29 (90.6) |  | 12 (100) | 32 (97.0) |  |
| N2 | 0 (0) | 0 (0) |  | 0 (0) | 0 (0) |  |
| N3 | 0 (0) | 0 (0) |  | 0 (0) | 0 (0) |  |
| Overall stage (8th edition) |  |  | 0.094 |  |  | 0.488 |
| III | 8 (61.5) | 10 (31.3) |  | 3 (25.0) | 14 (42.4) |  |
| IVA | 5 (38.5) | 22 (68.8) |  | 9 (75.0) | 19 (57.6) |  |
| IC regimen |  |  | 0.605 |  |  | 0.127 |
| TPF | 10 (76.9) | 28 (87.5) |  | 7 (58.3) | 25 (75.8) |  |
| TP | 1 (7.7) | 2 (6.3) |  | 1 (8.3) | 1 (3.0) |  |
| PF | 2 (15.4) | 0 (0) |  | 4 (33.3) | 3 (9.1) |  |
| GP | 0 (0) | 2 (6.3) |  | 0 (0) | 4 (12.1) |  |
| Concrrent chemotherapy cycles |  |  | 0.223 |  |  | 0.504 |
| 2 | 10 (76.9) | 29 (90.6) |  | 7 (58.3) | 21 (63.6) |  |
| 3 | 3 (23.1) | 3 (9.4) |  | 5 (41.7) | 12 (36.4) |  |

Note: Data are shown as number of patients (%).

Abbreviations: CR, complete response; PR, partial response; SD, stable disease; PD, disease progression; WHO, World Health Organization; IC, induction chemotherapy; TPF, docetaxel-cisplatin-5-fluorouracil; TP, docetaxel-cisplatin; PF, cisplatin-5-fluorouracil; GP, gemcitabine-cisplatin.

**Supplementary Figure Legends:**

**Figure S1:** Kaplan–Meier curves for OS (A), LRRFS (B), DMFS (C), and PFS (D) stratified by IC cycles in the whole cohort.

**Figure S2:** Kaplan–Meier curves for OS (A), LRRFS (B), DMFS (C), and PFS (D) stratified by IC cycles in the matched cohort.

**Figure S3:** Kaplan–Meier curves for OS (A), LRRFS (B), DMFS (C), and PFS (D) stratified by tumor response during IC in the whole cohort.

**Figure S4:** Kaplan–Meier curves for OS (A), LRRFS (B), DMFS (C), and PFS (D) stratified by IC cycles for N2-3 patients in the matched “SD/PD” group.

**Figure S5:** Kaplan–Meier curves for OS (A), LRRFS (B), DMFS (C), and PFS (D) stratified by IC cycles for N0-1 patients in the matched “CR/PR” group.

**Figure S6:** Kaplan–Meier curves for OS (A), LRRFS (B), DMFS (C), and PFS (D) stratified by IC cycles for N0-1 patients in the matched “SD/PD” group.
